# Further insights into the sialome switch of *Amblyomma americanum* adult females

**DOI:** 10.1186/s12864-026-12666-2

**Published:** 2026-03-16

**Authors:** Stephen Lu, Jose M.C. Ribeiro, Lucas Tirloni

**Affiliations:** 1 Tick-Pathogen Transmission Unit, Laboratory of Bacteriology, Division of Intramural Research, National Institute of Allergy and Infectious Diseases, Hamilton, MT USA; 2 Vector Biology Section, Laboratory of Malaria and Vector Research, Division of Intramural Research, National Institute of Allergy and Infectious Diseases, Rockville, MD USA

**Keywords:** tick, salivary glands, RNA-sequencing, vector, arthropod

## Abstract

**Background:**

The lone star tick, *Amblyomma americanum*, is a three-host species widely distributed across North America and a vector for multiple human pathogens. Bites from this tick is also associated with alpha-gal syndrome, underscoring its growing public health importance. Adult female ticks undergo a prolonged feeding cycle divided into preparatory, slow-feeding, and rapid-feeding phases. Tick salivary glands are essential for successful blood feeding, producing a complex mixture of molecules that modulate host hemostasis, immunity, and tissue repair. The dynamic remodeling of the salivary gland transcriptome and proteome, termed the sialome switch, is thought to support prolonged feeding and immune evasion. To investigate the molecular mechanisms underlying the sialome switch, we performed high-throughput transcriptomic profiling of salivary glands from *A. americanum* adult females across the feeding cycle.

**Results:**

Unlike previous studies that described the sialome switch in *A. americanum* by pooling ticks according to time post-attachment, we classified individuals based on average body weight, defining seven feeding stages encompassing unfed, slow-feeding, and rapid-feeding ticks. We identified 18,704 putative coding sequences (CDS) and revealed five distinct transcriptional profiles corresponding to different feeding stages. Unsupervised clustering indicated that most CDS were stage-specifically expressed, with temporal shifts observed in different protein families, including Kunitz-type, lipocalins, metalloproteases, and evasins. The transition from unfed to the slow-feeding phase corresponded to expression of a distinct subset of CDS within the signal transduction class, suggesting potential signaling pathways responsible for regulating this dynamic process.

**Conclusions:**

This study provides a high-resolution view of transcriptional dynamics in *A. americanum* salivary glands and identifies candidate molecular pathways regulating the sialome switch. These findings advance understanding of tick feeding biology and shed light on strategies ticks use to evade host defenses during prolonged blood meals.

**Supplementary Information:**

The online version contains supplementary material available at 10.1186/s12864-026-12666-2.

## Background

The lone star tick, *Amblyomma americanum*, is a three-host tick species that is widely distributed across North America, with specimens reported from 39 states and the District of Columbia [1, 2]. *A. americanum* can act as a vector for several pathogens that pose significant risks to human health, including *Francisella tularensis* [3], *Borrelia lonestari* [4], *Ehrlichia chaffeensis* [5], Heartland and Bourbon viruses [6]. Bites from this tick are also linked to the development of alpha-gal syndrome [7]. Given its extensive distribution and capacity to transmit a variety of harmful pathogens, the lone star tick represents an increasing public health concern.

The feeding cycle of the adult female ixodid tick is traditionally divided into three main phases. The first phase, known as the preparatory phase, involves attachment to the host and the establishment of the feeding lesion. The second phase, or slow-feeding phase, marks the onset of blood ingestion. This phase typically lasts several days, during which the female concentrates the blood meal and experiences a moderate weight gain. The final phase, called the rapid-feeding phase, occurs during the last 12 to 36 h of feeding. During this phase, the size and weight of the adult female increase exponentially, often exceeding 100 times the weight of an unfed adult tick [8]. The total weight gain achieved during a complete feeding cycle of adult females is unparalleled by any other blood-feeding arthropod, underscoring the distinctive hematophagic biology of ticks [8].

An essential component of blood feeding on a vertebrate host is the tick salivary gland. These glands produce saliva that contains a complex mixture of peptide and non-peptide molecules capable of modulating host hemostatic, immune, and tissue-repair responses. It has been nearly four decades since the first reports describing the antihemostatic, anti-inflammatory, and immunosuppressive activity of tick salivary proteins were published [9]. Since then, significant progress has been made in characterizing the pharmacological arsenal of tick salivary proteins and their potential role in blood acquisition [10]. It is now well established that, as feeding progresses, tick salivary glands undergo morphological changes [11, 12], accompanied by alterations in its protein composition [13, 14]. This phenomenon, unique to ticks, is known as sialome switch [15] and is hypothesized to support tick feeding on different hosts [16, 17] and to evade host defense responses mounted during their extended feeding period [15]. To date, the sialome switch has been examined primarily through transcriptomic and proteomic approaches of pooled ticks from different feeding stages [18–20], providing a partial understanding of this process.

In *Amblyomma americanum*, the sialome switch has been described through transcriptomic, proteomic, and immunoproteomic analyses [14, 19, 21], all of which relied on collecting ticks based on time of attachment. It is important to note, however, that the duration required for an adult female tick to achieve full engorgement varies considerably among species and hosts [22]. For example, *Ixodes ricinus* feeding on sheep complete their feeding cycle within 7 to 11 days. In contrast, *Haemaphysalis concinna* feeding on rabbits require 7 to 12 days, while *Dermacentor silvarum* feeding on both rabbits and guinea pigs, take between 6 and 12 days to fully engorge. In the rabbit model, *Ixodes scapularis* require 6 to 10 days, *Amblyomma americanum* 10 to 15 days, and *Dermacentor variabilis* 6 to 12 days [23]. These observations indicate that, at any given point during the feeding process, individual ticks may be at different stages of engorgement. Consequently, categorizing ticks solely by “days of feeding” or “time post-attachment” may introduce grouping bias.

Moreover, although substantial research has focused on identifying and characterizing tick salivary proteins, relatively few studies have investigated the molecular mechanisms that regulate the sialome switch. In this study, we conducted a high-throughput analysis of transcriptional changes in the salivary glands of *A. americanum* adult females throughout feeding. Rather than pooling ticks with varying feeding durations, we classified them based on average body weight, creating seven distinct groups comprising unfed ticks, slow-feeding ticks, and those in the rapid-feeding phase. Beyond providing a higher resolution view of the sialome switch, our aim was also to identify potential signaling pathways responsible for regulating this dynamic process.

## Methods

### Tick rearing and salivary gland dissection

*Amblyomma americanum* ticks were purchased from the tick rearing facility at Oklahoma State University (Stillwater, OK, USA). Unfed ticks were maintained at 21 °C and 80–90% relative humidity before infestation. Adult ticks used for salivary gland extraction were restricted to feed onto the outer part of the ear of four naïve female New Zealand White rabbits secured with an orthopedic stockinet. A total of 15 adult females and 15 males (30 ticks per ear, 60 ticks per animal) were placed into the tick containment apparatus and allowed to attach. To group ticks by a blood feeding index, partially fed ticks were collected from host during the feeding, selected based on their engorgement size, and sorted by their average weight in biological triplicates: group unfed (UF, 4.7 ± 0.62 mg, 10 ticks per sample), G1 (6.4 ± 0.60 mg, 5 ticks per sample), G2 (16.4 ± 1.82 mg, 5 ticks per sample), G3 (24.7 ± 3.24 mg, 5 ticks per sample), G4 (67.2 ± 7.30 mg, 5 ticks per sample), G5 (373.9 ± 34.48 mg, 3 ticks per sample), and G6 (577.0 ± 88.50 mg, 3 ticks per sample). After removal from the host, ticks were rinsed with 1% bleach, nuclease-free water, and 70% ethanol, followed by a final rinse with nuclease-free water. Ticks were dissected within two hours after removal from the host. Tick salivary glands (SGs) were dissected in a fresh, ice-cold nuclease-free phosphate-buffered saline (PBS), pH 7.4 (Invitrogen, Waltham, MA, USA). After dissection, SGs were gently washed in fresh nuclease-free PBS, pH 7.4, containing 4 U/mL of RNAse inhibitor (RNaseOUT, Thermo Fisher Scientific, Waltham, MA, USA) and a protease inhibitor cocktail (Sigma Aldrich, St. Louis, MO, USA). After washing, dissected SGs were immediately stored in RNAlater (Invitrogen, Waltham, MA, USA) until total RNA extraction.

### Library preparation, sequencing, and data analysis

Total RNA was isolated using the AllPrep DNA/RNA/Protein mini kit (QIAGEN, Germantown, MD, USA) according to the manufacturer`s instructions. RNA integrity and quantification were assessed using a 4200 TapeStation system (Agilent Technologies, Santa Clara, CA, USA). Due to poor RNA quality (RIN value lower than 7), one biological replicate of group 6 was removed from this study. The Illumina libraries were constructed using the NEBNextUltraTM II (Directional) RNA with polyA selection library prep kit and sequencing was performed in an Illumina Novaseq 6000 DNA sequencer. The quality of raw Illumina reads were checked using the FastQC tool (0.12.1) (https://www.bioinformatics.babraham.ac.uk/projects/fastqc/). Low-quality sequences with a Phred quality score (Q) below 20 and the Illumina adaptors were removed using TrimGalore (0.6.7) (https://github.com/FelixKrueger/TrimGalore). Subsequently, reads were merged and assembled *de novo* using Trinity (2.9.0) [24], in single-stranded F mode (forward read-pair orientation), and ABySS (2.3.6) [25] with k values ranging from 25 to 95, with increments of 10. The final assemblies were merged, and sequences sharing at least 95% identity were consolidated using the CD-HIT tool [26]. The DNA coding sequences (CDS) with an open reading frame (ORF) of at least 150 nucleotides were extracted based on BLASTp (2.2.30) results from several databases, including a subset of the non-redundant protein database, the transcriptome shotgun assembly (TSA), and RefSeq-invertebrate (https://www.ncbi.nlm.nih.gov/refseq). The CDS were extracted if they covered at least 70% of a matching protein. Additionally, all ORFs starting with a methionine and with a length of at least 40 amino acids were analyzed using the SignalP tool (V3.0). Sequences with a putative signal peptide were mapped to the ORFs, and the most 5` methionine was selected as the starting point of the transcript [27]. Relative quantification of each CDS was performed by mapping the trimmed Illumina reads to the final set of CDS using RSEM [28] and CDS with a TPM (transcripts per million) ≥ 3 in at least one biological condition were selected for downstream analysis. Functional annotation of the selected CDS was carried out using an *in-house* program that scanned a vocabulary of approximately 450 words and their order of appearance in the protein matches obtained from BLASTp/RPS-BLAST against various databases, including Transcriptome Shotgun Assembly (TSA), a subset from the Non-Redundant (NR), RefSeq-invertebrate, RefSeq-vertebrate, RefSeq-protozoa (https://www.ncbi.nlm.nih.gov/refseq/), UniProt Knowledgebase (UNIPROTKB - https://www.uniprot.org/help/uniprotkb), CDD, SMART, MEROPS, and PFAM [29]. This annotation process included percent identities and coverage information [30]. The databases utilized in this study were updated in December 2024. The final annotated CDS are available for download as a hyperlinked Excel file (Supplementary file 1). Transcriptome completeness was evaluated using the Benchmarking Universal Single-Copy Orthologs (BUSCO) utilizing the Arachnida database as reference [31].

### Statistical analysis

The multidimensional plot and the pairwise differential expression analysis were carried out with the edgeR package [32] for R [33]. Statistical significance was considered when Log_2_FoldChange (LogFC) was higher than 2 or less than − 2, and a false discovery rate (FDR) less than 0.05 were obtained. The heatmap plot was generated with the pheatmap package using the TPM values and the volcano plots were generated with the ggplot2 package for R. Unsupervised clustering of the filtered CDS, based on TPM values, were performed with the Expander tool using the CLICK method [34]. Ortholog detection was determined by the reciprocal smallest distance (RSD) method [35], run with a coverage ≥ 80% and an e-value ≤ 0.1.

### Data availability

The transcriptome data was deposited to the National Center for Biotechnology Information (NCBI) under BioProject PRJNA1225697 and BioSamples SAMN46899977 - SAMN46899996. The raw reads were deposited to the Short Reads Archive of the NCBI under accessions SRR32398692 - SRR32398711. This Transcriptome Shotgun Assembly project has been deposited at DDBJ/EMBL/GenBank under the accession GLLI00000000. The version described in this paper is the first version, GLLI01000000. The supplementary file 1 can be downloaded from https://proj-bip-prod-publicread.s3.us-east-1.amazonaws.com/transcriptome/Aamericanum_SG_2025/AaSg_SupData_1.zip.

## Results and discussion

### The sialome switch of *A. americanum* adult females

**Fig. 1:**
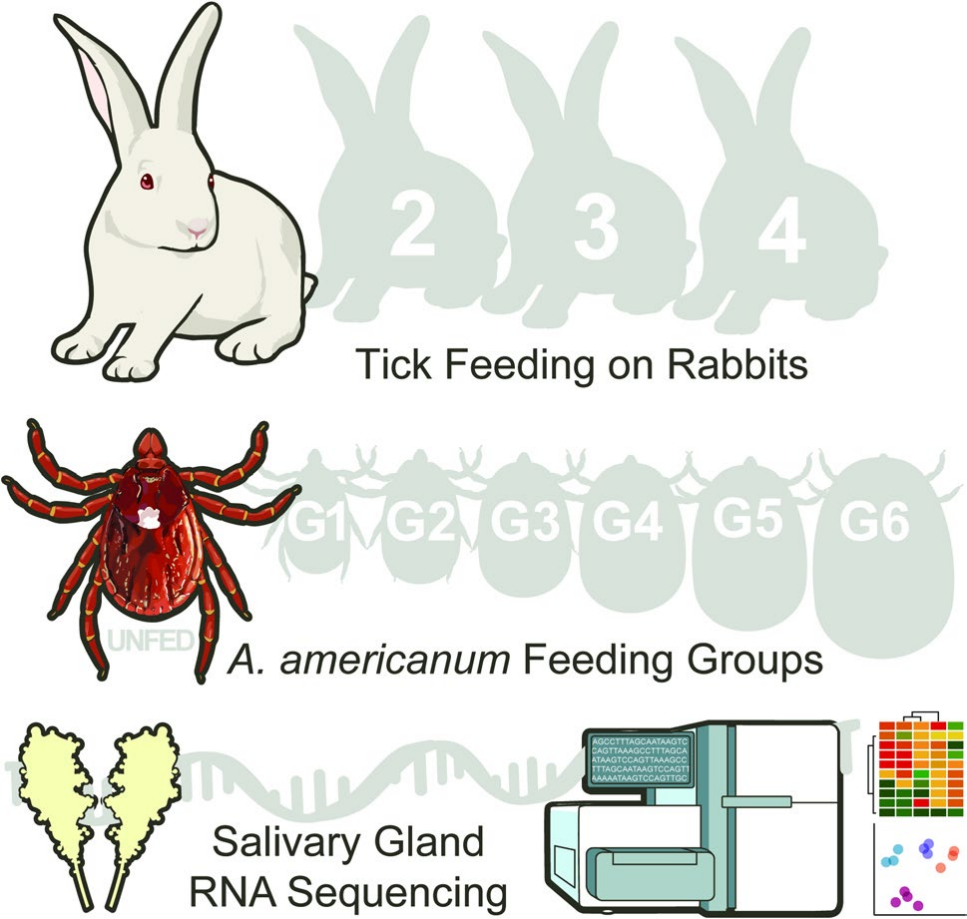
Graphical representation of the experimental design regarding *A. americanum* adult female feeding on rabbits

Based on the experimental design illustrated in the Fig. 1, Illumina-sequencing of the 20 libraries from *A. americanum* salivary glands collected at different feeding stages resulted in 1,096,404,568 high-quality reads, from which we identified 119,982 putative transcripts. To investigate the quality of our dataset, we employed the Benchmarking of Universal Single Copy Orthologs (BUSCO), which exhibited a completeness of 74.8% (62.1% single, 12.7% duplicated), 5.2% fragmented and 20% missing based on the Arachnida database.

Mapping of the trimmed Illumina reads to our putative CDS resulted in similar mapping rates across all samples (33.99% ± 6.54%, Sup. Table 1). It is important to note the overall high number of unmapped reads, approximately 66%, could be due to reads from 5’ and 3’ UTR regions that are removed during our CDS extraction step, reads from non-coding RNA or CDS that were not extract due to the lack of homology with previously deposited sequences, absence of a putative signal peptide or exhibited an open reading frame with less than 150 nucleotides. For downstream analysis we focused on the putative transcripts that presented an average TPM above three in at least one feeding stage, resulting in a final set of 18,704 putative CDS (Sup. File 1).

**Fig. 2:**
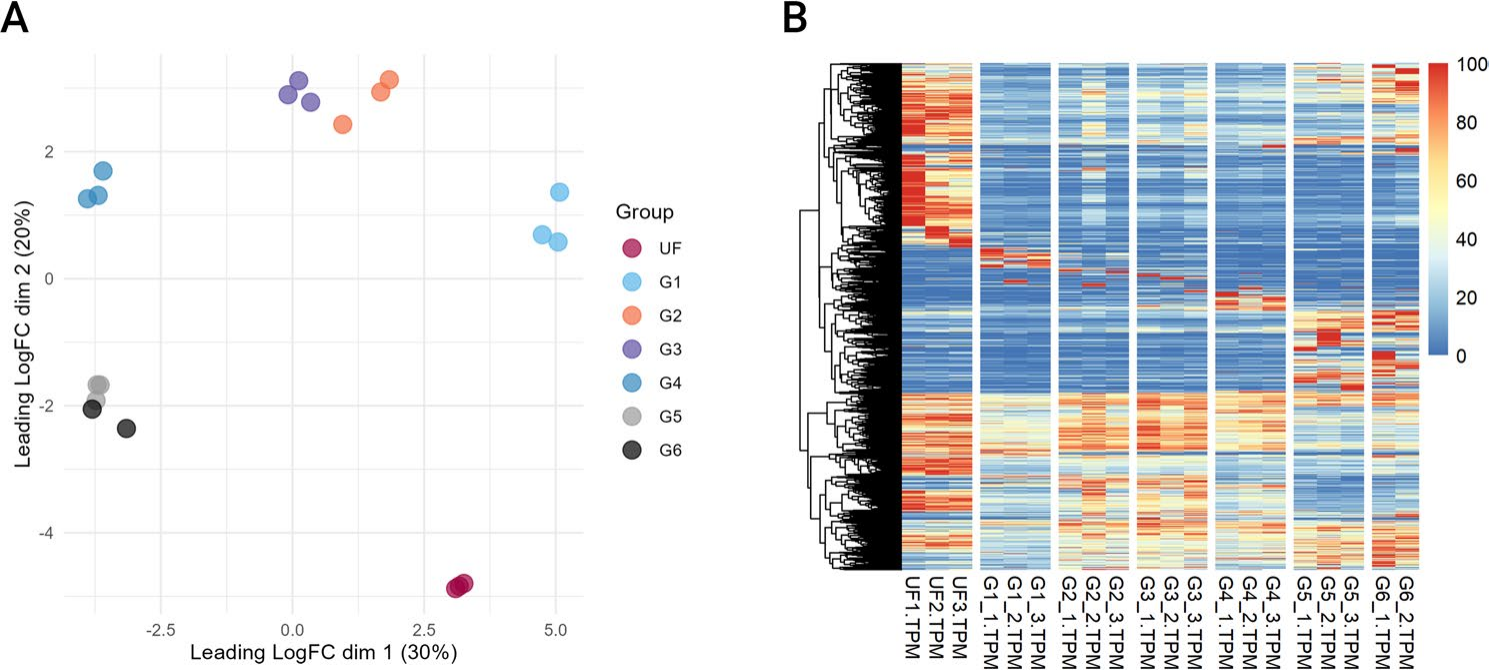
Overview of the sialome switch phenomenon in *A. americanum* adult females as feeding progresses. **A **Dimensional plot based on the TPM of the 18,704 putative transcripts that exhibited a TPM ≥ 3 in at least one biological condition. **B **Heatmap plot based on the normalized TPM of the 18,704 putative transcripts. The TPM values of each transcript can be found in the Supplementary file 1

A previous longitudinal sialome of *A. americanum* identified 5,792 putative CDS and defined four major transcriptional profiles based on expression patterns: unfed ticks, partially fed ticks attached for 12–48 h, partially fed ticks attached for 72–144 h, and ticks that had fed for 11 days [19]. In our study, we identified 18,704 putative CDS, greatly expanding the catalog of salivary gland transcripts. Our analysis also revealed five distinct transcriptional profiles (Fig. 2A). In addition to the four stages described previously – unfed (UF), attachment and beginning of feeding (G1), slow-feeding phase (G2 – G3) and rapid-feeding phase (G5 – G6), our approach also captured a distinct transition stage between slow and rapid-feeding (G4). Furthermore, the dimensionality reduction analysis revealed high consistency across all biological replicates, supporting average tick body weight as a reliable proxy for feeding stage classification [36, 37].

Exploration of transcript abundances across different feeding stages (Fig. 2B) provides further insights into the dynamics of the sialome switch phenomenon. For instance, several putative transcripts that are highly expressed in the salivary glands of unfed ticks are downregulated once feeding begins, remaining at low levels throughout the feeding cycle. In contrast, we identified transcripts that exhibit stage-specific expression, being predominantly abundant during either the slow- or rapid-feeding phases.

**Fig. 3:**
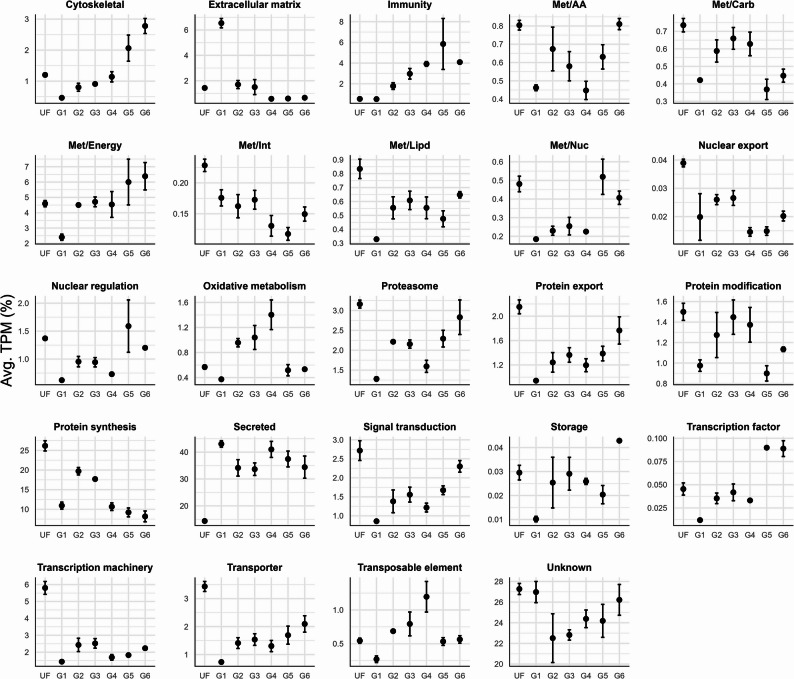
Functional annotation of the 18,704 putative transcripts with TPM ≥ 3 that were identified in the salivary glands of *A. americanum* adult females at different feeding stages. Dots represent the average TPM (as percentage) of the transcripts classified within each functional class. Error bars represent the standard error of the mean from the three biological replicates for each feeding stage

Functional annotation of the 18,704 putative transcripts into 24 functional classes (Fig. 3) revealed that the “secreted” functional group, comprising transcripts with a predicted signal peptide and potential salivary proteins involved in tick attachment and feeding, was among the most abundant class across all feeding stages, in alignment with previous sialome studies [19, 36]. Specifically, this class accounted for 14% of the total transcripts in unfed ticks and ranged from 33% to 43% in fed ticks. This suggests that unfed ticks possess a set of salivary proteins primed for attachment and the initiation of feeding. However, once feeding begins, there is a significant increase in salivary protein production that is maintained high throughout the feeding cycle, likely aiding in overcoming host defenses and supporting prolonged feeding.

It is important to acknowledge the challenges associated with functional annotation pipelines in non-model organisms. In our classification strategy, we included an “unknown” functional category, which accounted for approximately 20–28% of transcripts across all samples (Fig. 3). This category comprises transcripts that either share similarity with previously deposited sequences of unknown function or exhibit low to no similarity with known sequences. As such, these transcripts may represent potentially novel sequences and reflect the overall knowledge gap regarding the function of salivary proteins from ticks, emphasizing the need for further efforts to characterize these proteins.

**Fig. 4:**
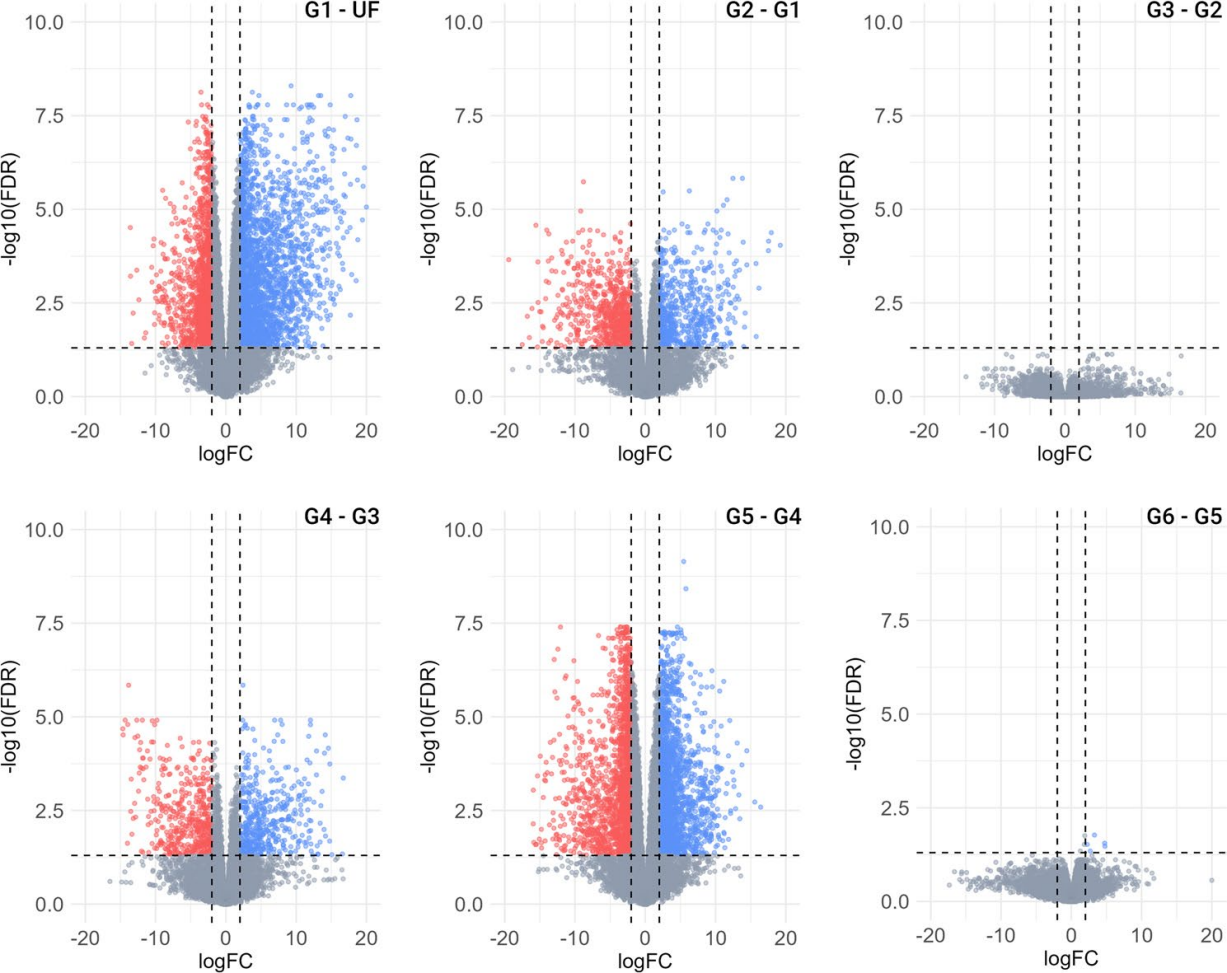
Volcano plots representing the differentially expressed transcripts identified in the salivary glands of *A. americanum* at different feeding stages. Transcripts were considered differentially expressed when a logFC ≥ 2 of logFC ≤ -2 (vertical lines), alongside an FDR < 0.05 (horizontal line) was obtained. Transcripts upregulated are represented as blue dots, transcripts downregulated are represented as red dots and those non-modulated as gray dots

**Fig. 5:**
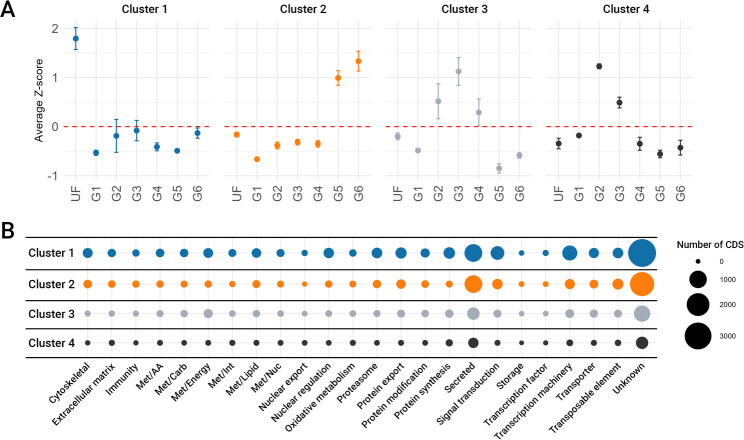
**A**: Unsupervised clustering of the 15,728 CDS based on their TPM in *A. americanum* salivary glands at different feeding stages. Dots represent the average Z-score of the TPM while error bars represent the standard deviation of the mean. **B** Bubble plot representing the functional annotation of the CDS identified within each cluster. The size of each sphere indicates the number of CDS

To systematically identify stage-specific transcripts, we performed differential expression analysis between specific feeding stages (Fig. 4) alongside unsupervised clustering of 18,704 putative transcripts based on their TPM values across different feeding stages (Fig. 5). The transition from unfed to fed ticks exhibited the highest number of modulated transcripts, 4,036 CDS (Fig. 4, G1 – UF). Indicating that attachment and beginning of feeding are major triggers for transcriptional regulation in tick salivary glands. When comparing the G2 – G1 groups, we identified 1,327 modulated transcripts, reflecting further adaptations in the salivary glands during the initial stages of feeding. However, as feeding progresses, no significant differences were observed between the comparison G3 – G2, indicating that the gene expression profile of the salivary glands becomes relatively stable during the slow-feeding stage. Additionally, the substantial number of modulated transcripts in the G4 – G3 and G5 – G4 comparisons, along with the differences in average weight between the groups (G3: 24.7 ± 3.24 mg, G4: 67.2 ± 7.30 mg, G5: 373.9 ± 34.48 mg), supports the notion that G4 represents a transitional stage between slow- and rapid-feeding phases. Interestingly, ticks in G4, with an average weight of 67.2 mg, may have reached the critical weight (~ 10-fold the weight of unfed ticks), as previously reported for *Amblyomma hebraeum* [38]. This critical weight is associated with the transition from the slow- to rapid-feeding phase and is accompanied by several behavioral and physiological changes, including alterations in hemolymph ecdysteroid titre, salivary gland degeneration, ovary weight, oocyte length, and oocyte vitellin content [38].

Similarly, our unsupervised approach clustered 15,728 CDS, approximately 84% of all CDS, into four clusters (Fig. 5A). Cluster 1, containing 7,973 CDS, was predominantly expressed in unfed ticks. Cluster 3 (2,098 CDS) and cluster 4 (772 CDS) showed increased expression during the slow-feeding stage, while cluster 2 (4,885 CDS) was enriched during the rapid-feeding phase. Functional annotation of the CDS in each cluster revealed a high abundance of transcripts classified as “unknown,” “secreted,” and “transcriptional machinery” (Fig. 5B).

Although informative, it is important to recognize the limitations of RNA-sequencing data, specifically that transcript abundance does not always correlate with protein abundance, highlighting the need for further proteomic validation. Similarly, additional validation of selected transcript abundances by qPCR is required. To our knowledge, McSwain and colleagues [13] were the first to investigate proteomic changes in the salivary glands of *A. americanum* adult females during feeding. In their seminal study, authors identified major differences in protein expression between unfed and partially fed ticks weighing 10 mg. Additional proteomic changes were observed between ticks weighing 10 mg and those reaching 100 mg. Findings from a more recent longitudinal proteomic study, which analyzed tick saliva composition every 24 h over the first eight days of feeding [14], revealed dynamic and progressive changes in protein profiles throughout the feeding process. Together, these proteomic studies provide partial validation for transcriptomic data presented here, despite the inherent limitations of RNA-based approaches.

To provide further insight into the sialome switch in *A. americanum* adult females, the following subsections offer an overview of the CDS enriched at each feeding stage, with a focus on their potential roles in blood-feeding and in driving the sialome switch.

### The transcriptional landscape of salivary glands of unfed ticks

To describe the salivary gland composition of unfed ticks we opted to focus on the unsupervised clustering data, which identify 7,973 putative CDS enriched in this stage (Fig. 5, cluster 1). The functional classification of such transcripts revealed that most of them fall into the “unknown”, “secreted”, “transcription machinery”, and “signal transduction” categories (Fig. 5B). However, based on their TPM values, the most abundant categories were “unknown”, “protein synthesis”, “secreted”, and “transcription machinery” (Fig. 3).

Within the “protein synthesis” category, we identified several CDS encoding putative ribosomal subunits among the most abundant transcripts in unfed ticks, with TPM values ranging from 4,527 to 11,186 (Sup. File 1). While in the “transcription machinery” category, we detected CDS encoding putative transcription initiation factors, helicases, and RNA-binding proteins, all showing consistently high expression levels (TPM > 1,000). In line with previous studies [18, 19], we also identified several CDS encoding putative secreted proteins, including lipocalin-like sequences, peptidases inhibitors from the Kunitz-type and cystatin families, 5’-nucleotidases, and metalloproteases [39]. Members of these protein families have been implicated in blood acquisition, specifically by modulating host immune [40, 41] and hemostatic [42, 43] responses. Also enriched in unfed ticks, were CDS coding putative glycine-rich proteins, which were shown to be key constituents of tick cement [44, 45]. Furthermore, the presence of such proteins has been confirmed to be present in the saliva and cement cone of *A. americanum* by mass spectrometry assays [14, 17, 44].

The overall abundance of transcripts related to “protein synthesis” and “transcription machinery” supports the idea that the salivary glands of unfed ticks are already transcriptionally active or primed for rapid protein production upon host attachment and the initiation of feeding, initially supported by the expression of these putative secreted proteins.

It is noteworthy that adult ticks can remain unfed for several days or weeks before encountering a suitable host. This raises an interesting question: are these abundant transcripts continuously transcribed throughout the unfed period, or are they produced early in the adult stage and maintained at high levels until feeding begins? Longitudinal *omics* studies focusing on unfed ticks will provide a better understanding regarding the stability, turnover and regulation of tick salivary glands transcripts.

Although the sialome switch phenomenon has been described in several tick species, the molecular mechanisms regulating this process remain unknown. Classical signaling pathways and epigenic events have been proposed as potential contributors [46]. Therefore, the “signal transduction” category is of particular interest, as it may shed light on these regulatory components. Notably, 497 putative CDS were assigned to this functional group, accounting for 2.7 ± 0.26% of the total TPM of unfed ticks. Furthermore, these CDS exhibited a distinct transcriptional profile, with higher expression levels in unfed ticks compared to fed individuals (Fig. 3).

Among the “signal transduction” CDS within cluster 1, we identified several components of classical signaling pathways. These included multiple sequences belonging to the seven-transmembrane G protein-coupled receptor (GPCR) superfamily (CDD: 14964: 7tm_GPCRs), which transduce extracellular signals – via binding of peptides, lipids, hormones, and neurotransmitters – into intracellular second messenger responses. The identified GPCRs could be further classified into three subfamilies: D2-like dopamine receptors, GAR-2 muscarinic acetylcholine receptors, and frizzled-like receptors, which are activated by the Wingless/Int-1 (WNT) lipoglycoproteins.

Previous research has shown that dopamine is a potent in vitro stimulant of fluid secretion in tick salivary glands [47]. Subsequent work identified a dopamine-sensitive adenylate cyclase, suggesting that cyclic AMP is likely the second messenger responsible for this secretory response [48]. Similarly, injection of pilocarpine, a muscarinic acetylcholine receptor agonist, is known to promote salivation in ticks [47], and more recent studies suggest a specific role for this signaling pathway in type I acini [49]. Together, these studies suggest the role of D2 dopamine and GAR-2 muscarinic receptors in tick salivation. In contrast, the function of frizzled-like receptors has not yet been specifically explored in the context of tick salivary gland physiology.

In addition to GPCRs, we identified multiple transcripts encoding calmodulin-like proteins, serine/threonine phosphatases and kinases from various subfamilies, including protein kinase C (PKC), Serine/Threonine kinase (STKc), Phosphoinositide-3-kinase (PI3K), and protein tyrosine kinase (PTKc). Furthermore, transcription factors belonging to the basic Helix-Loop-Helix (bHLH), basic Leucine Zipper Domain (BZIP), BTF3-like, HMG-Box, LHX and zinc-finger (ZF) were also enriched in the salivary glands of unfed tick. From an epigenetic perspective, multiple transcripts encoding putative histone acetyltransferases, deacetylases, demethylases and lysine-methyltransferases were also identified within cluster 1 (Sup. File 1).

The current understanding of signaling pathways and transcriptional regulation in ticks remains limited, with only a few kinases having been identified and functionally characterized to date. These included STKc members of the cyclin-dependent kinases (CDK) family, which play an important role in cell cycle regulation [50]; cAMP-dependent kinases associated with salivary secretion [51]; and ecdysteroid signaling, which contribute to salivary gland degeneration [52–54]. By contrast, although epigenetic mechanisms have been documented in ticks in responses to pathogen infection [55, 56] and embryogenesis [57], the role of histone modification in tick salivary glands, in the context of feeding, remains completely unexplored.

Nevertheless, considering that the transition from the unfed to the fed stage triggers major transcriptional changes in tick salivary glands (Fig. 2), and given the overall abundance of GPCRs, kinase-like sequences, and several transcription factors families in unfed ticks, as well as the presence of several putative enzymes capable of mediating histone post-translational modifications, it is likely that some of these components participate in regulating the sialome switch phenomenon. Overall, the identification of specific signaling pathways and potential epigenetic regulators within the salivary of ticks opens a largely unexplored field of research that may yield key insights into the molecular mechanism governing the tick sialome switch and could ultimately be leveraged to develop novel strategies for disrupting tick feeding. Targeted knockdown of specific genes and/or pharmacological inhibition of components of the pathways identified here, individually or in combination, would provide a more direct approach to testing the role of these pathways in the sialome switch phenomenon.

### The sialome of slow-feeding *A. americanum* adult females

The slow-feeding stage of adult female ticks spans multiple days, during which the tick concentrates its blood meal, resulting in a modest increase in body size and weight [37]. Although tick feeding is traditionally divided into three phases, clear distinction between the attachment phase and the onset of feeding remains challenging. Given the high number of differentially expressed transcripts identified between G1- UF and G2 – G1 (Fig. 4), we hypothesized that ticks in the G1 group may partially represent this transition phase and opted to include them in this section.

As previously mentioned, attachment and the initiation of blood feeding are major triggers of transcriptional changes in the tick salivary glands, as reflected by the highest number of modulated transcripts (Fig. 4, G1 – UF). Functional classification of these transcripts revealed that those associated with the “extracellular matrix” and “secreted” categories were the most upregulated, showing overall TPM ratio increases of 4.62- and 3.02-fold in G1 relative to UF, respectively. Notably, the “extracellular class” was found only upregulated in our G1 ticks, indicating a specific temporal regulation. In contrast, the most downregulated transcripts belonged to the “transcription machinery” (TPM ratio of 0.25) and “transporters” (TPM ratio of 0.21) functional classes (Sup. Table 2).

Within the upregulated CDS classified into the “extracellular matrix” functional group, we identified seven glycine-rich proteins, as well as putative cuticle proteins and GP1 cell wall proteins (Sup. File 1; Tab DEG_G1 – UF). Previous studies have reported multiple glycine-rich proteins in the cement of several tick species, including *A. americanum* [44], *Haemaphysalis flava* [58] and *Rhipicephalus microplus* [59], and have shown their upregulation in partially fed ticks compared to unfed ones. In addition to their role in cement formation and tick attachment, some glycine-rich proteins have been proposed to possess antimicrobial activity [60]. Given the pivotal role of cement in tick attachment, and consequently in feeding and pathogen transmission, several studies have evaluated glycine-rich proteins as vaccine candidates, with varying phenotypic outcomes such as reduced attachment rates, increased mortality, and decreased egg mass and hatch rates [61–63]. Notably, the “extracellular matrix” functional class was found specifically enriched within G1 ticks with all other feeding stages exhibiting expression levels similar to unfed ones (Fig. 3). Overall, this specific upregulation of CDS related to cement further supports the interpretation that the G1 group partially represents tick in the attachment phase and beginning of the slow-feeding phase.

Within the “secreted” class, the four most upregulated CDS (logFC 19–20) encoded putative Kunitz-type inhibitors (Sup. File 1). Notably, AaContig_166984 and ContigSigP-190588 maintained high TPM levels throughout the entire slow-feeding stage, whereas AaContig-214190 and ContigSigP-106321 showed peak expression at G1 (Sup. Figure 1). Also upregulated at G1 was a type 2 cystatin (AaContig_21303), a cysteine peptidase inhibitor. Tick salivary cystatins have been shown to modulate host immune response by interfering with cytokine production, T-cell proliferation, and leukocyte recruitment [64, 65], thereby creating a favorable environment for blood acquisition and pathogen transmission.

Previous omics studies have demonstrated that lipocalins are major components of the salivary glands in several tick species, including *A. americanum* [14, 19]. Corroborating with these findings, we identified multiple transcripts encoding putative lipocalins that were upregulated in both the G1 – UF and G2 – G1 comparisons (Sup. Figure 2). Tick lipocalins were initially characterized as high-affinity histamine-binding proteins [66] and as binders of other biogenic amines [67], thereby contributing to blood acquisition by interfering with host homeostatic responses. Subsequent studies described a lipocalin from the soft tick *Ornithodoros moubata* capable of binding fatty acids associated with inflammatory response and inhibiting complement activation through specific interactions with C5 [68], further expanding the proposed role of tick salivary lipocalins in successful blood-feeding.

The “signal transduction” functional class was among the most downregulated in the G1–UF comparison (TPM ratio of 0.32; Sup. Table 2), with several kinases, GTPases, and phosphatases showing logFC values between − 2 and − 6. The observed upregulation of classical signaling pathway components in unfed ticks, followed by their pronounced downregulation upon host attachment and feeding, suggests that these transcripts are maintained at high levels in unfed ticks to facilitate rapid molecular responses required for feeding initiation after host recognition. We hypothesize that, upon host recognition and/or attachment, these components are translated and activated, triggering major transcriptional shifts that determine the fate of tick salivary gland cells. Supporting this hypothesis is the upregulation of the “transcription factor” functional class in the G2–G1 comparison (TPM ratio of 2.97), likely reflecting activation of specific signaling pathways. Further proteomic and phosphoproteomic analyses will not only test this hypothesis but also clarify the specific pathway(s) involved in this process.

As feeding progresses, we observed further transcriptional changes within the salivary glands of *A. americanum* adult females, represented by G2 – G1 pairwise comparison (Sup. Table 3). Functionally, the highest number of modulated CDS belonged to the “secreted” group (585 transcripts), although the overall TPM levels were similar between both groups (TPM ratio of 0.79), whereas the second functional class with the highest number of modulated transcripts within the G2 – G1 comparison was the “unknown” group (488 CDS), underscoring the limited knowledge existing regarding tick salivary proteins.

Among the most upregulated and downregulated putative “secreted” CDS were those annotated as putative Kunitz-type and lipocalins (Sup. File 1). These findings highlight a defining aspect of the sialome switch phenomenon, in which different members of the same protein family are preferentially expressed at specific feeding stages (Sup. Figures 1 and 2). Given the extended feeding cycle of ticks, it was hypothesized that this temporal variation in salivary protein expression may function as a “smoke-and-screen” strategy against the host immune system. As the host mounts an effective immune response to antigens expressed during the early stages of feeding, the tick shifts to expressing alternative members of the same protein family, thereby evading immune recognition [15]. Further studies to determine whether these proteins possess redundant biochemical functions are needed, which would help elucidate the role of the sialome switch as an antigenic variation strategy that supports prolonged feeding on a vertebrate host.

Lastly, when comparing the G3 and G2 groups, no transcripts were found to be differentially expressed (Fig. 4). This suggests that, although the slow-feeding stage spans multiple days, the transcriptional landscape of *A. americanum* adult females remains relatively stable during this period. Similar findings have been reported in the salivary glands of *R. sanguineus* [36]. 

Overall, the data presented here provides a higher-resolution view of the sialome switch phenomenon. Specifically, our grouping strategy enabled the identification of an intermediate transcriptional state between unfed and slow-feeding ticks, representing the transition associated with host attachment and the onset of feeding. Characterizing the tick proteins enriched in this specific group is of considerable interest, as these molecules could serve as potential targets for disrupting tick feeding at its earliest stage. Moreover, the identification of specific enriched classical pathways provides a valuable foundation for elucidating the molecular mechanisms associated with the transcriptional regulation and the sialome switch. 

### The sialome of rapid-feeding *A. americanum* adult females

The rapid-feeding stage, also known as the “big sip,” occurs during the final 12–36 h of feeding and is characterized by the ingestion of a large volume of host blood, resulting in a dramatic increase in tick weight and size. In our dataset, this stage is represented by groups G5 and G6, which displayed nearly identical expression profiles, while ticks within group G4 exhibited an intermediate transcription profile (Fig. 2). Based on average tick weight and the substantial number of differentially expressed transcripts observed between G4–G3 and G5–G4, we propose that group G4 represents the transition from the slow- to the rapid-feeding stage (Fig. 4) and opted to include the G4 data in this section.

The transition from the slow- to the rapid-feeding stage, represented by the G4 – G3 and G5 – G4 pairwise comparisons, exhibited the second-highest number of modulated transcripts in our dataset, with 965 and 3,607 CDS, respectively (Fig. 4). These extensive transcriptional changes underscore the physiological shifts occurring in the salivary glands to accommodate the last feeding stage. Functional annotation of the differentially expressed transcripts between G4 – G3 and G5 – G4 revealed that the “secreted” class contained the largest number of modulated CDS (Sup. Tables 4 and 5), despite displaying relatively stable overall expression levels (TPM ratio of = 1.2). The second most modulated class was the “unknown”, with 344 (G4 – G3) and 1,556 (G5 – G4) transcripts. Notably, these two categories accounted for 65% and 61% of the total TPM in group G4 and G5, respectively, highlighting their abundance during this feeding period.

In the G4 – G3 comparison, the most upregulated transcripts in the “secreted” were those annotated putative lipocalins, metalloproteases, and Kunitz-type inhibitors. Among the most up-regulated CDS in the G5 – G4 comparison, we identified two putative metalloproteases belonging to the M12B subfamilies as the most strongly modulated transcripts. AaContig_198533 and AaContig_26069 displayed logFC values of 16.4 and 15.5, respectively. Metalloproteases are known to be among the most abundant salivary proteins in feeding ticks across multiple species [14, 19, 36, 69]. Consistent with other secreted proteins, we observed several putative metalloproteases that were differentially regulated at specific feeding stages (Sup. Figure 3), suggesting that members of this protein family are expressed throughout the full feeding cycle of adult females.

Functionally, tick salivary metalloproteases have been shown to degrade gelatin, fibrinogen, and fibronectin, thereby disrupting host homeostatic responses triggered by tick attachment and feeding [70, 71]. Recent work further demonstrates that rabbits can mount antibody responses against salivary metalloproteases, and that inhibition of these enzymes reduces tick engorgement weight at feeding completion. Notably, this phenotype was not observed in artificial feeding experiments, indicating that metalloproteases likely act on host-derived components that are absent from artificial feeding systems [72]. Together, these studies underscore the importance of salivary metalloproteases in blood acquisition. Their high abundance, larger number or paralogs, and stage-specific expression patterns all point to a major functional role in feeding. It is also important to note that only a fraction of these metalloproteases has been functionally characterized to date. Thus, additional, yet-undiscovered roles for members of this family in the context of blood acquisition remain possible.

Also enriched in the salivary glands of fast-feeding ticks was a putative evasin-like (ContigSigp-103596), which reached an average TPM exceeding 15,000 in G5 (Sup. Figure 4). Evasins constitute a large family of tick salivary proteins that can be classified into three main groups – A1, A2, and B – based on their primary structure features and their CC- or CXC-chemokine-binding activity [73]. Functionally, the first evasin to be characterized was evasin-1 from *Rhipicephalus sanguineus*, which binds the CC chemokines CCL3, CCL4, and CCL18 [74, 75]. Subsequent work revealed additional specificity profiles within the family: evasin-3 binds CXCL1 and CXCL8, whereas evasin-4 binds CCL5 and CCL11 [76]. Because chemokines play central roles in cell recruitment, immune responses, and homeostasis, their neutralization facilitates prolonged tick feeding. The high abundance of evasins and the presence of multiple paralogs in tick salivary glands suggest an ability to simultaneously neutralize diverse chemokines, thereby reducing the inflammatory responses at the tick bite site.

Lastly, virtually no difference in transcript expression was observed between G6 and G5 (Fig. 4), supporting the notion that the salivary transcriptional landscape of fast-feeding ticks is largely conserved. Nevertheless, examining the corresponding protein profiles is still necessary to confirm this hypothesis. Although advances in omics technologies and the availability of high-quality tick genomic data enabled us to assemble and identify potential CDS, many remain functionally uncharacterized, as reflected by the high prevalence of “unknown” annotations across all feeding stages. Continued characterization efforts will facilitate the discovery of novel pharmacological activities and mechanisms employed by tick salivary glands to support their unique hematophagic behavior, ultimately informing new strategies for tick control.

## Conclusion

In the current study, we present a longitudinal transcriptomic analysis of the salivary glands of *A. americanum* adult females across different feeding stages. The data provides a higher-resolution view of the sialome switch, highlighting distinct expression patterns of several salivary proteins whose activities are associated with blood feeding. Moreover, our analysis identifies components of classical signaling pathways as well as epigenetic regulators in tick salivary glands that may contribute to regulating the sialome switch. It is well established that some tick-borne pathogens require a defined transmission window. Therefore, understanding the mechanisms that govern the sialome switch is critical, as it may reveal targets for disrupting both tick feeding and pathogen transmission Future longitudinal proteomic and phosphoproteomic studies will further elucidate the molecular mechanisms underlying this phenomenon and may ultimately support the development of novel anti-tick strategies.

## Supplementary Information

Supplementary Material 1.

## Data Availability

The transcriptome data was deposited to the National Center for Biotechnology Information (NCBI) under BioProject PRJNA1225697 and BioSamples SAMN46899977 - SAMN46899996. The raw reads were deposited to the Short Reads Archive of the NCBI under accessions SRR32398692 - SRR32398711. This Transcriptome Shotgun Assembly project has been deposited at DDBJ/EMBL/GenBank under the accession GLLI00000000. The version described in this paper is the first version, GLLI01000000. The supplementary file 1 can be downloaded from [https://proj-bip-prod-publicread.s3.us-east-1.amazonaws.com/transcriptome/Aamericanum\_SG\_2025/AaSg\_SupData\_1.zip.

## Abbreviations

ABySSa: Assembly by short sequencesa
BLASTpa: Basic local alignment search tool for proteinsa
BLASTp: Basic local alignment search tool for proteinsa
BUSCO: Benchmarking universal single-copy orthologs
bHLH: Basic helix–loop–helix domain
bZIP: Basic leucine zipper domain
CCL: CC chemokine ligand
CD-HIT: Cluster database at high identity with tolerance
CDK: Cyclin-dependent kinase
CDS: Coding sequences
CDD: Conserved domain database
CLICK: Cluster identification via connectivity kernels
CXCL: CXC chemokine ligand
DDBJ: DNA data bank of Japan
DEG: Differentially expressed genes
edgeR: Empirical analysis of digital gene expression data in R
EMBL: European Molecular Biology Laboratory
FDR: False discovery rate
GAR-2: Muscarinic acetylcholine receptor subtype 2
GPCR: G protein–coupled receptor
HMG: High-mobility group
k: k-mer length
LogFC: Log2 fold change
LHX: LIM homeobox domain
MEROPS: Peptidase database
NCBI: National Center for Biotechnology Information
NR: Non-redundant protein database
ORF: Open reading frame
PBS: Phosphate-buffered saline
PFAM: Protein families database
PI3K: Hosphoinositide 3-kinase
PKC: Protein kinase C
PTKc: Protein tyrosine kinase catalytic domain
RIN: RNA integrity number
RNA: Ribonucleic acid
RNase: Ribonuclease
RPS-BLAST: Reverse position-specific BLAST
RSD: Reciprocal smallest distance
RSEM: RNA-seq by expectation maximization
SG: Salivary glands
SMART: Simple modular architecture research tool
STKc: Serine/threonine kinase catalytic domain
TPM: Transcripts per million
TSA: Transcriptome shotgun assembly
UF: Unfed
UniProtKB: UniProt Knowledgebase
UTR: Untranslated region
ZF: Zinc-finger domain

## References

[1] Springer YP, Eisen L, Beati L, James AM, Eisen RJ. Spatial distribution of counties in the continental United States with records of occurrence of *Amblyomma americanum* (Ixodida: Ixodidae). J Med Entomol. 2014;51(2):342–51. 24724282 10.1603/me13115 PMC4623429

[2] SonenshineDE. Range Expansion of Tick Disease Vectors in North America: Implications for Spread of Tick-Borne Disease. Int J Environ Res Public Health. 2018;15(3). 10.3390/ijerph15030478. 10.3390/ijerph15030478 PMC5877023 29522469

[3] Hopla CE, Downs CM. The isolation of Bacterium tularense from the tick, *Amblyomma americanum*. J Kansas Entomol Soc. 1953;26(2):72–3.

[4] James AM, Liveris D, Wormser GP, Schwartz I, Montecalvo MA, Johnson BJ. Borrelia lonestari infection after a bite by an *Amblyomma americanum* tick. J Infect Dis. 2001;183(12):1810–4. 11372036 10.1086/320721

[5] Anderson BE, Sims KG, Olson JG, Childs JE, Piesman JF, Happ CM, Maupin GO, Johnson BJ. *Amblyomma americanum*: a potential vector of human ehrlichiosis. Am J Trop Med Hyg. 1993;49(2):239–44. 8357086 10.4269/ajtmh.1993.49.239

[6] Dupuis AP 2nd, Lange RE, Ciota AT. Emerging tickborne viruses vectored by *Amblyomma americanum* (Ixodida: Ixodidae): Heartland and Bourbon viruses. J Med Entomol. 2023;60(6):1183–96. 37862097 10.1093/jme/tjad060

[7] Sharma SR, Crispell G, Mohamed A, Cox C, Lange J, Choudhary S, Commins SP, Karim S. Alpha-Gal Syndrome: Involvement of *Amblyomma americanum* alpha-D-Galactosidase and beta-1,4 Galactosyltransferase Enzymes in alpha-Gal Metabolism. Front Cell Infect Microbiol. 2021;11:775371. 34926322 10.3389/fcimb.2021.775371 PMC8671611

[8] Soneshine DE, Roe RM. Biology of Ticks. 2nd ed. Oxford University Press; 2013.

[9] Ribeiro JM, Makoul GT, Levine J, Robinson DR, Spielman A. Antihemostatic, antiinflammatory, and immunosuppressive properties of the saliva of a tick, *Ixodes dammini*. J Exp Med. 1985;161(2):332–44. 2982989 10.1084/jem.161.2.332 PMC2187567

[10] Francischetti IM, Sa-Nunes A, Mans BJ, Santos IM, Ribeiro JM. The role of saliva in tick feeding. Front Biosci (Landmark Ed). 2009;14(6):2051–88. 19273185 10.2741/3363 PMC2785505

[11] Binnington KC. Sequential changes in salivary gland structure during attachment and feeding of the cattle tick, *Boophilus microplus*. Int J Parasitol. 1978;8(2):97–115. 681074 10.1016/0020-7519(78)90004-8

[12] Megaw MJW, Beadle DJ. Structure and Function of the Salivary-Glands of the Tick, *Boophilus-Microplus* Canestrini (Acarina, Ixodidae). Int J Insect Morphol. 1979;8(2):67–83.

[13] McSwain JL, Essenberg RC, Sauer JR. Protein changes in the salivary glands of the female lone star tick, *Amblyomma americanum*, during feeding. J Parasitol. 1982;68(1):100–6. 7077436

[14] Kim TK, Tirloni L, Pinto AFM, Diedrich JK, Moresco JJ, Yates JR 3rd, Vaz daS, Mulenga I Jr. Time-resolved proteomic profile of *Amblyomma americanum* tick saliva during feeding. PLoS Negl Trop Dis. 2020;14(2):e0007758. 32049966 10.1371/journal.pntd.0007758 PMC7041860

[15] Ribeiro JMC, Mans BJ. TickSialoFam (TSFam): A Database That Helps to Classify Tick Salivary Proteins, a Review on Tick Salivary Protein Function and Evolution, With Considerations on the Tick Sialome Switching Phenomenon. Front Cell Infect Microbiol. 2020;10:374. 32850476 10.3389/fcimb.2020.00374 PMC7396615

[16] Narasimhan S, Booth CJ, DePonte K, Wu MJ, Liang X, Mohanty S, Kantor F, Fikrig E. Host-specific expression of Ixodes scapularis salivary genes. Ticks Tick Borne Dis. 2019;10(2):386–97. 30545615 10.1016/j.ttbdis.2018.12.001

[17] Tirloni L, Kim TK, Pinto AFM, Yates JR 3rd, da Silva Vaz I Jr., Mulenga A. Tick-Host Range Adaptation: Changes in Protein Profiles in Unfed Adult *Ixodes scapularis* and *Amblyomma americanum* Saliva Stimulated to Feed on Different Hosts. Front Cell Infect Microbiol. 2017;7:517. 29312895 10.3389/fcimb.2017.00517 PMC5742094

[18] Aljamali MN, Hern L, Kupfer D, Downard S, So S, Roe BA, Sauer JR, Essenberg RC. Transcriptome analysis of the salivary glands of the female tick *Amblyomma americanum* (Acari: Ixodidae). Insect Mol Biol. 2009;18(2):129–54. 19320755 10.1111/j.1365-2583.2009.00863.x

[19] KarimS, Ribeiro JMC. An Insight into the Sialome of the Lone Star Tick, with a Glimpse on Its Time Dependent Gene Expression. PLoS ONE. 2015;10(7). 10.1371/journal.pone.0131292. 10.1371/journal.pone.0131292 PMC4489193 26131772

[20] Ribeiro JM, Francischetti IM. Role of arthropod saliva in blood feeding: sialome and post-sialome perspectives. Annu Rev Entomol. 2003;48:73–88. 12194906 10.1146/annurev.ento.48.060402.102812

[21] Radulovic ZM, Kim TK, Porter LM, Sze SH, Lewis L, Mulenga A. A 24–48 h fed *Amblyomma americanum* tick saliva immuno-proteome. BMC Genomics. 2014;15:518. 24962723 10.1186/1471-2164-15-518 PMC4099483

[22] Balashov YS. Bloodsucking Ticks (Ixodoidea)—Vectors of Diseases of Man and Animals. 1st. ed. Nauka Publishers; 1968.

[23] Troughton DR, Levin ML. Life cycles of seven ixodid tick species (Acari: Ixodidae) under standardized laboratory conditions. J Med Entomol. 2007;44(5):732–40. 17915502 10.1603/0022-2585(2007)44[732:lcosit]2.0.co;2

[24] Grabherr MG, Haas BJ, Yassour M, Levin JZ, Thompson DA, Amit I, Adiconis X, Fan L, Raychowdhury R, Zeng Q, et al. Full-length transcriptome assembly from RNA-Seq data without a reference genome. Nat Biotechnol. 2011;29(7):644–52. 21572440 10.1038/nbt.1883 PMC3571712

[25] Simpson JT, Wong K, Jackman SD, Schein JE, Jones SJ. Birol I: ABySS: a parallel assembler for short read sequence data. Genome Res. 2009;19(6):1117–23. 19251739 10.1101/gr.089532.108 PMC2694472

[26] Fu L, Niu B, Zhu Z, Wu S, Li W. CD-HIT: accelerated for clustering the next-generation sequencing data. Bioinformatics. 2012;28(23):3150–2. 23060610 10.1093/bioinformatics/bts565 PMC3516142

[27] Bendtsen JD, Nielsen H, von Heijne G, Brunak S. Improved prediction of signal peptides: SignalP 3.0. J Mol Biol. 2004;340(4):783–95. 15223320 10.1016/j.jmb.2004.05.028

[28] LiB, Dewey CN. RSEM: accurate transcript quantification from RNA-Seq data with or without a reference genome. BMC Bioinformatics 2011;12. 10.1186/1471-2105-12-323. 10.1186/1471-2105-12-323 PMC3163565 21816040

[29] Marchler-Bauer A, Derbyshire MK, Gonzales NR, Lu S, Chitsaz F, Geer LY, Geer RC, He J, Gwadz M, Hurwitz DI, et al. CDD: NCBI’s conserved domain database. Nucleic Acids Res. 2015;43(Database issue):D222–226. 25414356 10.1093/nar/gku1221 PMC4383992

[30] KarimS, Singh P, Ribeiro JMC. A Deep Insight into the Sialotranscriptome of the Gulf Coast Tick, *Amblyomma maculatum*. PLoS ONE. 2011;6(12). 10.1371/journal.pone.0028525. 10.1371/journal.pone.0028525 PMC3244413 22216098

[31] Simao FA, Waterhouse RM, Ioannidis P, Kriventseva EV, Zdobnov EM. BUSCO: assessing genome assembly and annotation completeness with single-copy orthologs. Bioinformatics. 2015;31(19):3210–2. 26059717 10.1093/bioinformatics/btv351

[32] Robinson MD, McCarthy DJ, Smyth GK. edgeR: a Bioconductor package for differential expression analysis of digital gene expression data. Bioinformatics. 2010;26(1):139–40. 19910308 10.1093/bioinformatics/btp616 PMC2796818

[33] R Core Team. R: A Language and Environment for Statistical Computing. R Foundation for Statistical Computing, Vienna, Austria; 2025. https://www.R-project.org/.

[34] Shamir R, Maron-Katz A, Tanay A, Linhart C, Steinfeld I, Sharan R, Shiloh Y, Elkon R. EXPANDER–an integrative program suite for microarray data analysis. BMC Bioinformatics. 2005;6:232. 16176576 10.1186/1471-2105-6-232 PMC1261157

[35] Wall DP, Fraser HB, Hirsh AE. Detecting putative orthologs. Bioinformatics. 2003;19(13):1710–1. 15593400 10.1093/bioinformatics/btg213

[36] Tirloni L, Lu S, Calvo E, Sabadin G, Di Maggio LS, Suzuki M, Nardone G, da Silva Vaz I Jr., Ribeiro JMC. Integrated analysis of sialotranscriptome and sialoproteome of the brown dog tick *Rhipicephalus sanguineus* (s.l.): Insights into gene expression during blood feeding. J Proteom. 2020;229:103899. 10.1016/j.jprot.2020.103899 PMC9514304 32673754

[37] Lu S, de Sousa-Paula LC, Ribeiro JMC, Tirloni L. Exploring the longitudinal expression dynamics of midguts in adult female *Amblyomma americanum* ticks. BMC Genomics. 2024;25(1):996. 39448894 10.1186/s12864-024-10905-y PMC11515579

[38] Weiss BL, Reuben Kaufman W. The relationship between ‘critical weight’ and 20-hydroxyecdysone in the female ixodid tick, *Amblyomma hebraeum*. J Insect Physiol. 2001;47(11):1261–7. 12770177 10.1016/s0022-1910(01)00112-3

[39] Rawlings ND, Barrett AJ, Thomas PD, Huang X, Bateman A, Finn RD. The MEROPS database of proteolytic enzymes, their substrates and inhibitors in 2017 and a comparison with peptidases in the PANTHER database. Nucleic Acids Res. 2018;46(D1):D624–32. 29145643 10.1093/nar/gkx1134 PMC5753285

[40] Kotsyfakis M, Sa-Nunes A, Francischetti IM, Mather TN, Andersen JF, Ribeiro JM. Antiinflammatory and immunosuppressive activity of sialostatin L, a salivary cystatin from the tick *Ixodes scapularis*. J Biol Chem. 2006;281(36):26298–307. 16772304 10.1074/jbc.M513010200

[41] Zavasnik-Bergant T, Vidmar R, Sekirnik A, Fonovic M, Salat J, Grunclova L, Kopacek P, Turk B. Salivary Tick Cystatin OmC2 Targets Lysosomal Cathepsins S and C in Human Dendritic Cells. Front Cell Infect Microbiol. 2017;7:288. 28713775 10.3389/fcimb.2017.00288 PMC5492865

[42] Francischetti IM, Valenzuela JG, Andersen JF, Mather TN, Ribeiro JM. Ixolaris, a novel recombinant tissue factor pathway inhibitor (TFPI) from the salivary gland of the tick, *Ixodes scapularis*: identification of factor X and factor Xa as scaffolds for the inhibition of factor VIIa/tissue factor complex. Blood. 2002;99(10):3602–12. 11986214 10.1182/blood-2001-12-0237

[43] Stutzer C, Mans BJ, Gaspar AR, Neitz AW, Maritz-Olivier C. *Ornithodoros savignyi*: soft tick apyrase belongs to the 5’-nucleotidase family. Exp Parasitol. 2009;122(4):318–27. 19393241 10.1016/j.exppara.2009.04.007

[44] Hollmann T, Kim TK, Tirloni L, Radulovic ZM, Pinto AFM, Diedrich JK, Yates JR 3rd, da, Silva Vaz I, Mulenga A Jr. Identification and characterization of proteins in the Amblyomma americanum tick cement cone. Int J Parasitol. 2018, 48(3–4):211–224. 29258831 10.1016/j.ijpara.2017.08.018 PMC5844823

[45] Mulenga A, Radulovic Z, Porter L, Britten TH, Kim TK, Tirloni L, Gaithuma AK, Adeniyi-Ipadeola GO, Dietrich JK, Moresco JJ, et al. Identification and characterization of proteins that form the inner core *Ixodes scapularis* tick attachment cement layer. Sci Rep. 2022;12(1):21300. 36494396 10.1038/s41598-022-24881-4 PMC9734129

[46] Perner J, Kropackova S, Kopacek P, Ribeiro JMC. Sialome diversity of ticks revealed by RNAseq of single tick salivary glands. PLoS Negl Trop Dis. 2018;12(4):e0006410. 29652888 10.1371/journal.pntd.0006410 PMC5919021

[47] Kaufman W. The influence of various factors on fluid secretion by in vitro salivary glands of ixodid Ticks. J Exp Biol. 1976;64(3):727–42. 180228 10.1242/jeb.64.3.727

[48] Schmidt SP, Essenberg RC, Sauer JR. A dopamine sensitive adenylate cyclase in the salivary glands of *Amblyomma americanum* (L). Comp Biochem Physiol C Comp Pharmacol. 1982;72(1):9–14. 6125338 10.1016/0306-4492(82)90197-6

[49] Mateos-Hernandez L, Defaye B, Vancova M, Hajdusek O, Sima R, Park Y, Attoui H, Simo L. Cholinergic axons regulate type I acini in salivary glands of *Ixodes ricinus* and *Ixodes scapularis* ticks. Sci Rep. 2020;10(1):16054. 32994503 10.1038/s41598-020-73077-1 PMC7524744

[50] Gomes H, Romeiro NC, Braz GR, de Oliveira EA, Rodrigues C, da Fonseca RN, Githaka N, Isezaki M, Konnai S, Ohashi K, et al. Identification and structural-functional analysis of cyclin-dependent kinases of the cattle tick *Rhipicephalus (Boophilus) microplus*. PLoS ONE. 2013;8(10):e76128. 24146826 10.1371/journal.pone.0076128 PMC3795742

[51] Palmer MJ, McSwain JL, Spatz MD, Tucker JS, Essenberg RC, Sauer JR. Molecular cloning of cAMP-dependent protein kinase catalytic subunit isoforms from the lone star tick, *Amblyomma americanum* (L). Insect Biochem Mol Biol. 1999;29(1):43–51. 10070744 10.1016/s0965-1748(98)00103-9

[52] Mao H, McBlain WA, Kaufman WR. Some properties of the ecdysteroid receptor in the salivary gland of the ixodid tick, *Amblyomma hebraeum*. Gen Comp Endocrinol. 1995;99(3):340–8. 8536946 10.1006/gcen.1995.1118

[53] Hu S, Wang Y, Xu Z, Zhou Y, Cao J, Zhang H, Zhou J. Identification of the Bcl-2 and Bax homologs from *Rhipicephalus haemaphysaloides* and their function in the degeneration of tick salivary glands. Parasit Vectors. 2021;14(1):386. 34348769 10.1186/s13071-021-04879-z PMC8336254

[54] Lu X, Zhang Z, Yuan D, Zhou Y, Cao J, Zhang H, da Silva Vaz I Jr., Zhou J. The ecdysteroid receptor regulates salivary gland degeneration through apoptosis in *Rhipicephalus haemaphysaloides*. Parasit Vectors. 2021;14(1):612. 34930413 10.1186/s13071-021-05052-2 PMC8686549

[55] Cabezas-Cruz A, Alberdi P, Ayllon N, Valdes JJ, Pierce R, Villar M, de la Fuente J. *Anaplasma phagocytophilum* increases the levels of histone modifying enzymes to inhibit cell apoptosis and facilitate pathogen infection in the tick vector *Ixodes scapularis*. Epigenetics. 2016;11(4):303–19. 27019326 10.1080/15592294.2016.1163460 PMC4889282

[56] MacIntosh GH, Nuyens AC, Vickery JL, Berthold A, Lloyd VK. Epigenetic responses in Borrelia-infected *Ixodes scapularis* ticks: Over-expression of euchromatic histone lysine methyltransferase 2 and no change in DNA methylation. PLoS ONE. 2025;20(6):e0324546. 40472016 10.1371/journal.pone.0324546 PMC12140222

[57] AmaranteAM, de Oliveira DM, de Souza M, Fonseca-Oliveira M, Galina A, Rosignoli S, Arcanjo AF, Moraes B, Paiardini A, Rotili D et al. Unraveling the Roles of Epigenetic Regulators During the Embryonic Development of *Rhipicephalus microplus*. Int J Mol Sci. 2025;26(18). 10.3390/ijms26189171. 10.3390/ijms26189171 PMC12470784 41009732

[58] Liu L, Cheng R, Mao SQ, Duan DY, Feng LL, Cheng TY. Saliva proteome of partially- and fully-engorged adult female *Haemaphysalis flava* ticks. Vet Parasitol. 2023;318:109933. 37043866 10.1016/j.vetpar.2023.109933

[59] Leal BF, Alzugaray MF, Seixas A, Da Silva Vaz I, Ferreira CAS. Characterization of a glycine-rich protein from *Rhipicephalus microplus*: tissue expression, gene silencing and immune recognition. Parasitology. 2018;145(7):927–38. 29144218 10.1017/S0031182017001998

[60] Alekseev AN, Burenkova LA, Podboronov VM, Chunikhin SP. Bacteriocidal qualities of ixodid tick (Acarina: Ixodidae) salivary cement plugs and their changes under the influence of a viral tick-borne pathogen. J Med Entomol. 1995;32(5):578–82. 7473610 10.1093/jmedent/32.5.578

[61] Mulenga A, Sugimoto C, Sako Y, Ohashi K, Musoke A, Shubash M, Onuma M. Molecular characterization of a *Haemaphysalis longicornis* tick salivary gland-associated 29-kilodalton protein and its effect as a vaccine against tick infestation in rabbits. Infect Immun. 1999;67(4):1652–8. 10084999 10.1128/iai.67.4.1652-1658.1999 PMC96509

[62] Harnnoi T, Watchabunsook S, Sakaguchi T, Xuan X, Fujisaki K. Characterization of *Haemaphysalis longicornis* recombinant cement-like antigens and preliminary study of their vaccination effects. J Vet Med Sci. 2006;68(12):1289–95. 17213697 10.1292/jvms.68.1289

[63] Zhou J, Gong H, Zhou Y, Xuan X, Fujisaki K. Identification of a glycine-rich protein from the tick *Rhipicephalus haemaphysaloides* and evaluation of its vaccine potential against tick feeding. Parasitol Res. 2006;100(1):77–84. 16802136 10.1007/s00436-006-0243-7

[64] Sa-Nunes A, Bafica A, Antonelli LR, Choi EY, Francischetti IM, Andersen JF, Shi GP, Chavakis T, Ribeiro JM, Kotsyfakis M. The immunomodulatory action of sialostatin L on dendritic cells reveals its potential to interfere with autoimmunity. J Immunol. 2009;182(12):7422–9. 19494265 10.4049/jimmunol.0900075 PMC2694955

[65] Kotal J, Stergiou N, Busa M, Chlastakova A, Berankova Z, Rezacova P, Langhansova H, Schwarz A, Calvo E, Kopecky J, et al. The structure and function of Iristatin, a novel immunosuppressive tick salivary cystatin. Cell Mol Life Sci. 2019;76(10):2003–13. 30747251 10.1007/s00018-019-03034-3 PMC11105445

[66] Paesen GC, Adams PL, Harlos K, Nuttall PA, Stuart DI. Tick histamine-binding proteins: isolation, cloning, and three-dimensional structure. Mol Cell. 1999;3(5):661–71. 10360182 10.1016/s1097-2765(00)80359-7

[67] Mans BJ, Ribeiro JM, Andersen JF. Structure, function, and evolution of biogenic amine-binding proteins in soft ticks. J Biol Chem. 2008;283(27):18721–33. 18445596 10.1074/jbc.M800188200 PMC2441560

[68] Roversi P, Lissina O, Johnson S, Ahmat N, Paesen GC, Ploss K, Boland W, Nunn MA, Lea SM. The structure of OMCI, a novel lipocalin inhibitor of the complement system. J Mol Biol. 2007;369(3):784–93. 17445829 10.1016/j.jmb.2007.03.064 PMC2724154

[69] Guizzo MG, Mans B, Pienaar R, Ribeiro JMC. A comparison of Illumina and PacBio methods to build tick salivary gland transcriptomes confirms large expression of lipocalins and other salivary protein families that are not represented in available tick genomes. Ticks Tick Borne Dis. 2023;14(6):102209. 37327738 10.1016/j.ttbdis.2023.102209 PMC10527494

[70] Francischetti IM, Mather TN, Ribeiro JM. Cloning of a salivary gland metalloprotease and characterization of gelatinase and fibrin(ogen)lytic activities in the saliva of the Lyme disease tick vector *Ixodes scapularis*. Biochem Biophys Res Commun. 2003;305(4):869–75. 12767911 10.1016/s0006-291x(03)00857-x PMC2903890

[71] Decrem Y, Beaufays J, Blasioli V, Lahaye K, Brossard M, Vanhamme L, Godfroid E. A family of putative metalloproteases in the salivary glands of the tick *Ixodes ricinus*. FEBS J. 2008;275(7):1485–99. 18279375 10.1111/j.1742-4658.2008.06308.x

[72] Perner J, Helm D, Haberkant P, Hatalova T, Kropackova S, Ribeiro JM, Kopacek P. The Central Role of Salivary Metalloproteases in Host Acquired Resistance to Tick Feeding. Front Cell Infect Microbiol. 2020;10:563349. 33312963 10.3389/fcimb.2020.563349 PMC7708348

[73] Bhattacharya S, Nuttall PA. Phylogenetic Analysis Indicates That Evasin-Like Proteins of Ixodid Ticks Fall Into Three Distinct Classes. Front Cell Infect Microbiol. 2021;11:769542. 34746035 10.3389/fcimb.2021.769542 PMC8569228

[74] Frauenschuh A, Power CA, Deruaz M, Ferreira BR, Silva JS, Teixeira MM, Dias JM, Martin T, Wells TNC, Proudfoot AEI. Molecular cloning and characterization of a highly selective chemokine-binding protein from the tick *Rhipicephalus sanguineus*. J Biol Chem. 2007;282(37):27250–8. 17640866 10.1074/jbc.M704706200

[75] Dias JM, Losberger C, Deruaz M, Power CA, Proudfoot AE, Shaw JP. Structural basis of chemokine sequestration by a tick chemokine binding protein: the crystal structure of the complex between Evasin-1 and CCL3. PLoS ONE. 2009;4(12):e8514. 20041127 10.1371/journal.pone.0008514 PMC2796168

[76] Deruaz M, Frauenschuh A, Alessandri AL, Dias JM, Coelho FM, Russo RC, Ferreira BR, Graham GJ, Shaw JP, Wells TN, et al. Ticks produce highly selective chemokine binding proteins with antiinflammatory activity. J Exp Med. 2008;205(9):2019–31. 18678732 10.1084/jem.20072689 PMC2526197

