# Supplementary material for: Further insights into the sialome switch of Amblyomma americanum adult females
Source: BMC Genomics. 2026 Mar 16;27:411. doi: 10.1186/s12864-026-12666-2 (PMC13113040; doi:10.1186/s12864-026-12666-2)
Supplement: Supplementary file 1 — Supplementary Material 1. [file 12864_2026_12666_MOESM1_ESM.docx]

**Supplementary Table 1:** Mapping rates of the trimmed Illumina reads to the putative CDS extracted from the *de novo* assembly of *A. americanum* salivary glands.

| Sample | No. of reads not aligned | No. of reads aligned | No. of total reads | No. of reads aligned (%) |
| --- | --- | --- | --- | --- |
| UF_1 | 34,783,858 | 23,677,118 | 58,460,976 | 40.50 |
| UF_2 | 28,761,509 | 17,943,911 | 46,705,420 | 38.42 |
| UF_3 | 38,256,682 | 24,188,597 | 62,445,279 | 38.73 |
|  |  |  |  |  |
| G1_1 | 33,901,471 | 22,054,581 | 55,956,052 | 39.41 |
| G1_2 | 28,871,584 | 19,699,584 | 48,571,168 | 40.56 |
| G1_3 | 32,037,168 | 20,603,274 | 52,640,442 | 39.14 |
|  |  |  |  |  |
| G2_1 | 50,353,900 | 26,508,471 | 76,862,371 | 34.49 |
| G2_2 | 25,180,858 | 16,721,753 | 41,902,611 | 39.91 |
| G2_3 | 31,046,578 | 17,708,442 | 48,755,020 | 36.32 |
|  |  |  |  |  |
| G3_1 | 46,370,591 | 25,825,264 | 72,195,855 | 35.77 |
| G3_2 | 34,989,949 | 19,744,755 | 54,734,704 | 36.07 |
| G3_3 | 31,741,821 | 21,137,160 | 52,878,981 | 39.97 |
|  |  |  |  |  |
| G4_1 | 27,298,878 | 14,387,722 | 41,686,600 | 34.51 |
| G4_2 | 31,369,858 | 16,367,391 | 47,737,249 | 34.29 |
| G4_3 | 31,447,207 | 15,402,994 | 46,850,201 | 32.88 |
|  |  |  |  |  |
| G5_1 | 42,108,187 | 14,217,275 | 56,325,462 | 25.24 |
| G5_2 | 41,980,452 | 10,987,710 | 52,968,162 | 20.74 |
| G5_3 | 34,821,127 | 11,324,742 | 46,145,869 | 24.54 |
|  |  |  |  |  |
| G6_1 | 49,865,569 | 14,053,734 | 63,919,303 | 21.99 |
| G6_2 | 50,555,781 | 18,107,062 | 68,662,843 | 26.37 |

**Supplementary table 2:** Functional annotation of the differentially expressed transcripts between G1 and UF groups.

| Class | Down  Regulated | Up  Regulated | Non-modulated | TPM UF | TPM G1 | TPM G1/UF |
| --- | --- | --- | --- | --- | --- | --- |
| Cytoskeletal | 34 | 11 | 259 | 11825.56 | 4583.02 | 0.39 |
| **Extracellular matrix** | **30** | **23** | **134** | **14069.9** | **64950.6** | **4.62** |
| Immunity | 15 | 14 | 83 | 5293.43 | 5127.59 | 0.97 |
| Met/AA | 18 | 27 | 157 | 7915.73 | 4585.32 | 0.58 |
| Met/Carb | 16 | 31 | 157 | 7242.85 | 4177.84 | 0.58 |
| Met/Energy | 17 | 38 | 318 | 45234.38 | 23855.6 | 0.53 |
| Met/Int | 5 | 21 | 102 | 2249.03 | 1744.19 | 0.78 |
| Met/Lipd | 32 | 32 | 231 | 8216.9 | 3261.89 | 0.4 |
| Met/Nuc | 7 | 27 | 120 | 4735.19 | 1833.06 | 0.39 |
| Nuclear export | 1 | 3 | 29 | 383.4 | 197.08 | 0.51 |
| Nuclear regulation | 36 | 23 | 239 | 13489.08 | 6174.31 | 0.46 |
| Oxidative metabolism | 8 | 27 | 92 | 5594.48 | 3700.6 | 0.66 |
| Proteasome | 46 | 17 | 307 | 31123.91 | 12695.35 | 0.41 |
| Protein export | 50 | 26 | 431 | 21196.56 | 9351.55 | 0.44 |
| Protein modification | 22 | 35 | 206 | 14770.1 | 9674.46 | 0.66 |
| Protein synthesis | 22 | 30 | 413 | 257624.9 | 108474 | 0.42 |
| **Secreted** | **250** | **834** | **2607** | **141337.9** | **427326.8** | **3.02** |
| Signal transduction | 128 | 31 | 658 | 26756.99 | 8503.01 | 0.32 |
| Storage | 0 | 1 | 18 | 290.91 | 100.94 | 0.35 |
| Transcription factor | 4 | 2 | 22 | 447.12 | 118.01 | 0.26 |
| Transcription machinery | 97 | 41 | 872 | 57135.95 | 14266.32 | 0.25 |
| Transporter | 63 | 61 | 296 | 33840.73 | 7262.72 | 0.21 |
| Transposable element | 28 | 88 | 556 | 5362.94 | 2688.12 | 0.5 |
| Unknown | 627 | 978 | 6582 | 268534.8 | 267690.5 | 1 |

**TPM:** Transcript per million

**
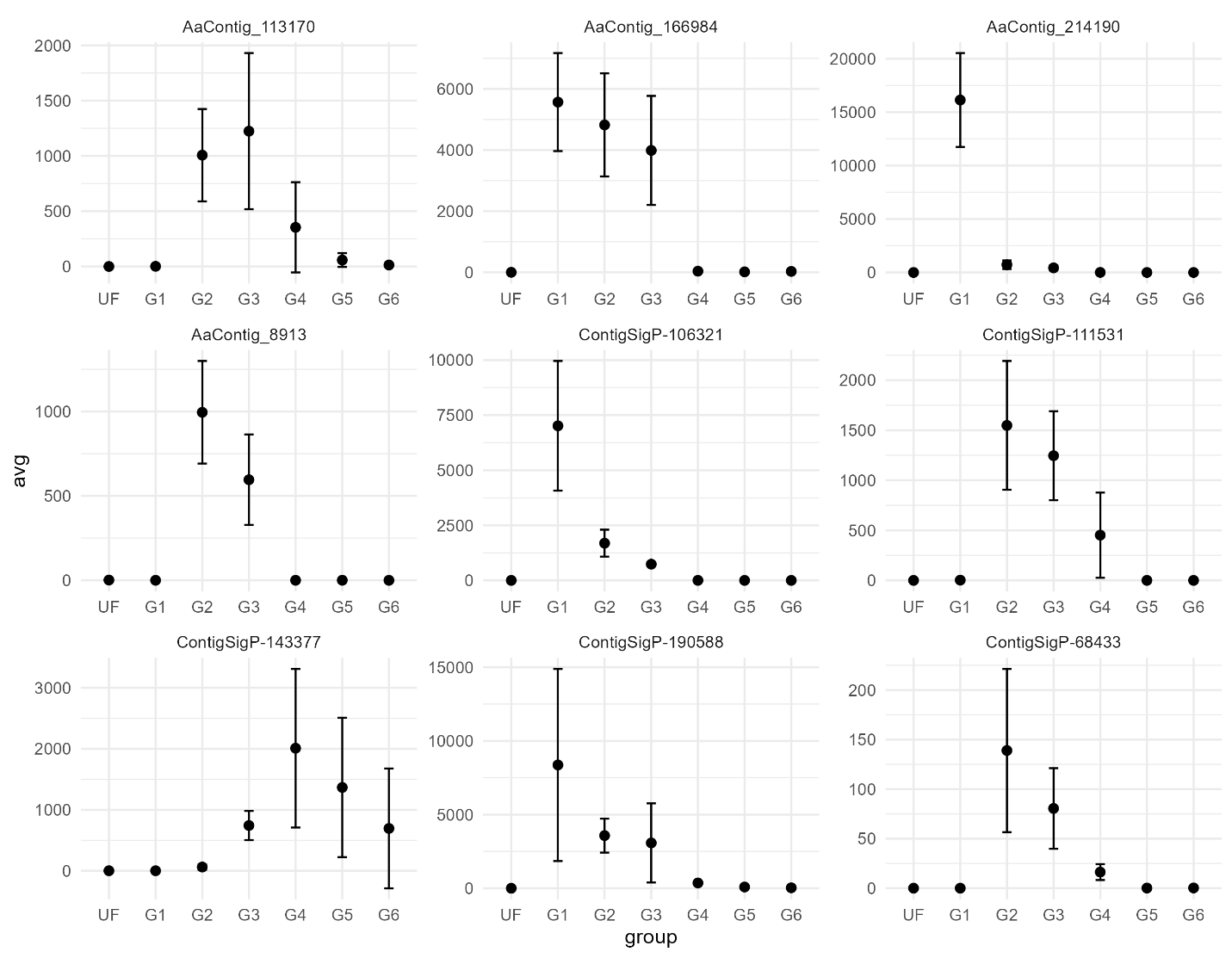
**

**Supplementary figure 1:** Expression profile of selected Kunitz-type inhibitors found upregulated in the early feeding stages in the salivary glands of *A. americanum* adult female ticks. Dots represent the average TPM and error bars the standard deviation of the mean.


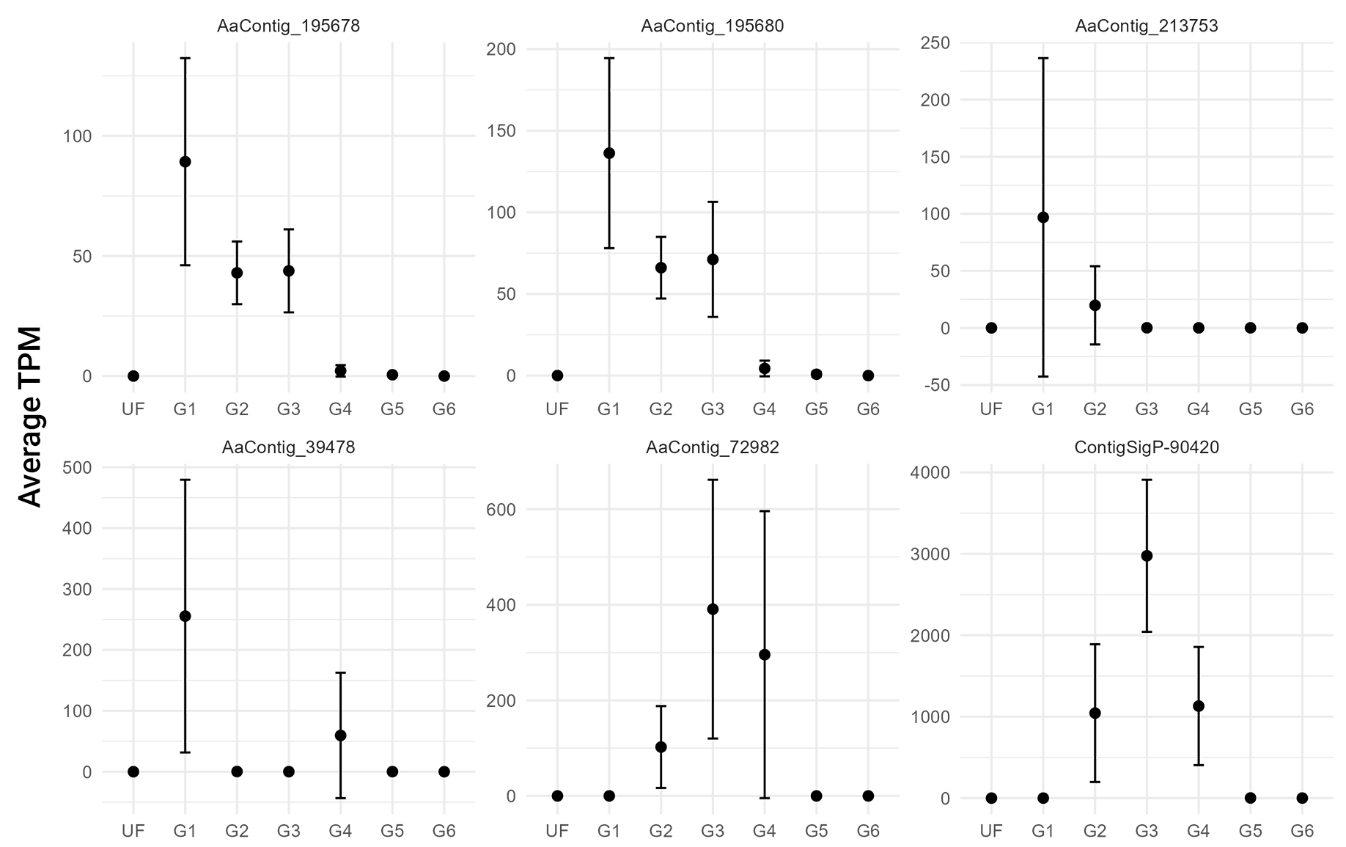


**Supplementary figure 2:** Expression profile of selected lipocalin-like transcripts found differentially expressed in the salivary glands of *A. americanum* adult females at different feeding stages. Dots represent the average TPM and error bars the standard deviation of the mean.

**Supplementary table 3:** Functional annotation of the differentially expressed transcripts between G2 and G1 groups.

| Class | Down  regulated | Up  regulated | Non-modulated | G1 TPM | G2 TPM | TPM G2/G1 |
| --- | --- | --- | --- | --- | --- | --- |
| Cytoskeletal | 2 | 1 | 301 | 4583.02 | 7929.37 | 1.73 |
| Extracellular matrix | 16 | 10 | 161 | 64950.6 | 16820.47 | 0.26 |
| Immunity | 8 | 6 | 98 | 5127.593 | 17612.52 | 3.43 |
| Met/AA | 10 | 9 | 183 | 4585.32 | 6674.137 | 1.46 |
| Met/Carb | 3 | 4 | 197 | 4177.84 | 5820.293 | 1.39 |
| Met/Energy | 12 | 3 | 358 | 23855.6 | 44625.54 | 1.87 |
| Met/Int | 3 | 2 | 123 | 1744.19 | 1607.187 | 0.92 |
| Met/Lipd | 7 | 8 | 280 | 3261.89 | 5490.79 | 1.68 |
| Met/Nuc | 6 | 0 | 148 | 1833.057 | 2272.43 | 1.24 |
| Nuclear export | 0 | 0 | 33 | 197.08 | 257.51 | 1.31 |
| Nuclear regulation | 1 | 2 | 295 | 6174.31 | 9450.163 | 1.53 |
| Oxidative metabolism | 5 | 5 | 117 | 3700.6 | 9477.357 | 2.56 |
| Proteasome | 1 | 1 | 368 | 12695.35 | 21928.71 | 1.73 |
| Protein export | 5 | 2 | 500 | 9351.55 | 12297.01 | 1.31 |
| Protein modification | 5 | 2 | 256 | 9674.46 | 12611.34 | 1.3 |
| Protein synthesis | 0 | 5 | 460 | 108474 | 195160.4 | 1.8 |
| **Secreted** | **328** | **257** | **3106** | **427326.8** | **338256.7** | **0.79** |
| Signal transduction | 6 | 15 | 796 | 8503.01 | 13667.07 | 1.61 |
| Storage | 0 | 1 | 18 | 100.9433 | 251.2867 | 2.49 |
| Transcription factor | 0 | 1 | 27 | 118.0133 | 349.9367 | 2.97 |
| Transcription machinery | 5 | 2 | 1003 | 14266.32 | 24043.25 | 1.69 |
| Transporter | 4 | 28 | 388 | 7262.717 | 13976.53 | 1.92 |
| Transposable element | 27 | 21 | 624 | 2688.117 | 6798.963 | 2.53 |
| Unknown | 266 | 222 | 7699 | 267690.5 | 222896.2 | 0.83 |

**TPM:** Transcript per million

**Supplementary table 4:** Functional annotation of the differentially expressed transcripts between G4 and G3.

| Class | Down  regulated | Up  Regulated | Non-  Modulated | G3 TPM | G4 TPM | TPM (G4/G3) |
| --- | --- | --- | --- | --- | --- | --- |
| Cytoskeletal | 3 | 4 | 297 | 8985.253 | 11193.93 | 1.25 |
| Extracellular matrix | 14 | 2 | 171 | 14788 | 5620.507 | 0.38 |
| Immunity | 4 | 5 | 103 | 29313.52 | 38521.73 | 1.31 |
| Met/AA | 7 | 2 | 193 | 5727.583 | 4396.923 | 0.77 |
| Met/Carb | 7 | 2 | 195 | 6518.787 | 6172.917 | 0.95 |
| Met/Energy | 1 | 3 | 369 | 46559.83 | 44610.3 | 0.96 |
| Met/Int | 3 | 0 | 125 | 1706.347 | 1281.97 | 0.75 |
| Met/Lipd | 9 | 6 | 280 | 6005.66 | 5447.7 | 0.91 |
| Met/Nuc | 4 | 5 | 145 | 2512.87 | 2213.6 | 0.88 |
| Nuclear export | 1 | 0 | 32 | 262.53 | 143.44 | 0.55 |
| Nuclear regulation | 1 | 0 | 297 | 9322.89 | 7176.247 | 0.77 |
| Oxidative metabolism | 10 | 4 | 113 | 10280.89 | 13801.19 | 1.34 |
| Proteasome | 0 | 2 | 368 | 21302.08 | 15671.28 | 0.74 |
| Protein export | 2 | 3 | 502 | 13477.04 | 11752.95 | 0.87 |
| Protein modification | 5 | 4 | 254 | 14309.63 | 13497.15 | 0.94 |
| Protein synthesis | 1 | 0 | 464 | 174918.5 | 104760.6 | 0.6 |
| Secreted | 242 | 188 | 3261 | 332775.7 | 403464.8 | 1.21 |
| Signal transduction | 7 | 3 | 807 | 15383.1 | 11952.49 | 0.78 |
| Storage | 1 | 0 | 18 | 287.3033 | 254.3767 | 0.89 |
| Transcription factor | 0 | 1 | 27 | 413.0533 | 325.31 | 0.79 |
| Transcription machinery | 3 | 1 | 1006 | 24886.91 | 16616.66 | 0.67 |
| Transporter | 13 | 8 | 399 | 15199.14 | 12821.81 | 0.84 |
| Transposable element | 6 | 34 | 632 | 7838.877 | 11758.64 | 1.5 |
| Unknown | 142 | 202 | 7843 | 225518.8 | 239670.9 | 1.06 |

**TPM:** Transcripts per million

**Supplementary table 5:** Functional annotation of the differentially expressed transcripts between G5 and G4

| Class | Down  regulated | Up  regulated | Non-modulated | G4 TPM | G5 TPM | TPM (G5/G4) |
| --- | --- | --- | --- | --- | --- | --- |
| Cytoskeletal | 11 | 23 | 270 | 11193.93 | 20325.24 | 1.82 |
| Extracellular matrix | 16 | 22 | 149 | 5620.507 | 5841.57 | 1.04 |
| Immunity | 8 | 19 | 85 | 38521.73 | 57636.68 | 1.5 |
| Met/AA | 32 | 14 | 156 | 4396.923 | 6215.453 | 1.41 |
| Met/Carb | 44 | 15 | 145 | 6172.917 | 3626.037 | 0.59 |
| Met/Energy | 51 | 16 | 306 | 44610.3 | 59122.57 | 1.33 |
| Met/Int | 17 | 7 | 104 | 1281.97 | 1156.427 | 0.9 |
| Met/Lipd | 21 | 27 | 247 | 5447.7 | 4686.03 | 0.86 |
| Met/Nuc | 17 | 14 | 123 | 2213.6 | 5117.717 | 2.31 |
| Nuclear export | 2 | 2 | 29 | 143.44 | 146.5533 | 1.02 |
| Nuclear regulation | 23 | 9 | 266 | 7176.247 | 15657.65 | 2.18 |
| Oxidative metabolism | 17 | 14 | 96 | 13801.19 | 5098.47 | 0.37 |
| Proteasome | 11 | 21 | 338 | 15671.28 | 22575.61 | 1.44 |
| Protein export | 41 | 39 | 427 | 11752.95 | 13660.56 | 1.16 |
| Protein modification | 28 | 18 | 217 | 13497.15 | 8856.123 | 0.66 |
| Protein synthesis | 44 | 10 | 411 | 104760.6 | 90742.05 | 0.87 |
| **Secreted** | **403** | **454** | **2834** | **403464.8** | **368974.5** | **0.91** |
| Signal transduction | 61 | 72 | 684 | 11952.49 | 16483.18 | 1.38 |
| Storage | 2 | 1 | 16 | 254.3767 | 200.6933 | 0.79 |
| Transcription factor | 3 | 5 | 20 | 325.31 | 885.7567 | 2.72 |
| Transcription machinery | 83 | 53 | 874 | 16616.66 | 17980.15 | 1.08 |
| Transporter | 56 | 51 | 313 | 12821.81 | 16679.39 | 1.3 |
| Transposable element | 52 | 101 | 519 | 11758.64 | 5255.95 | 0.45 |
| **Unknown** | **662** | **894** | **6631** | **239670.9** | **238231.1** | **0.99** |

**TPM:** Transcript per million

**
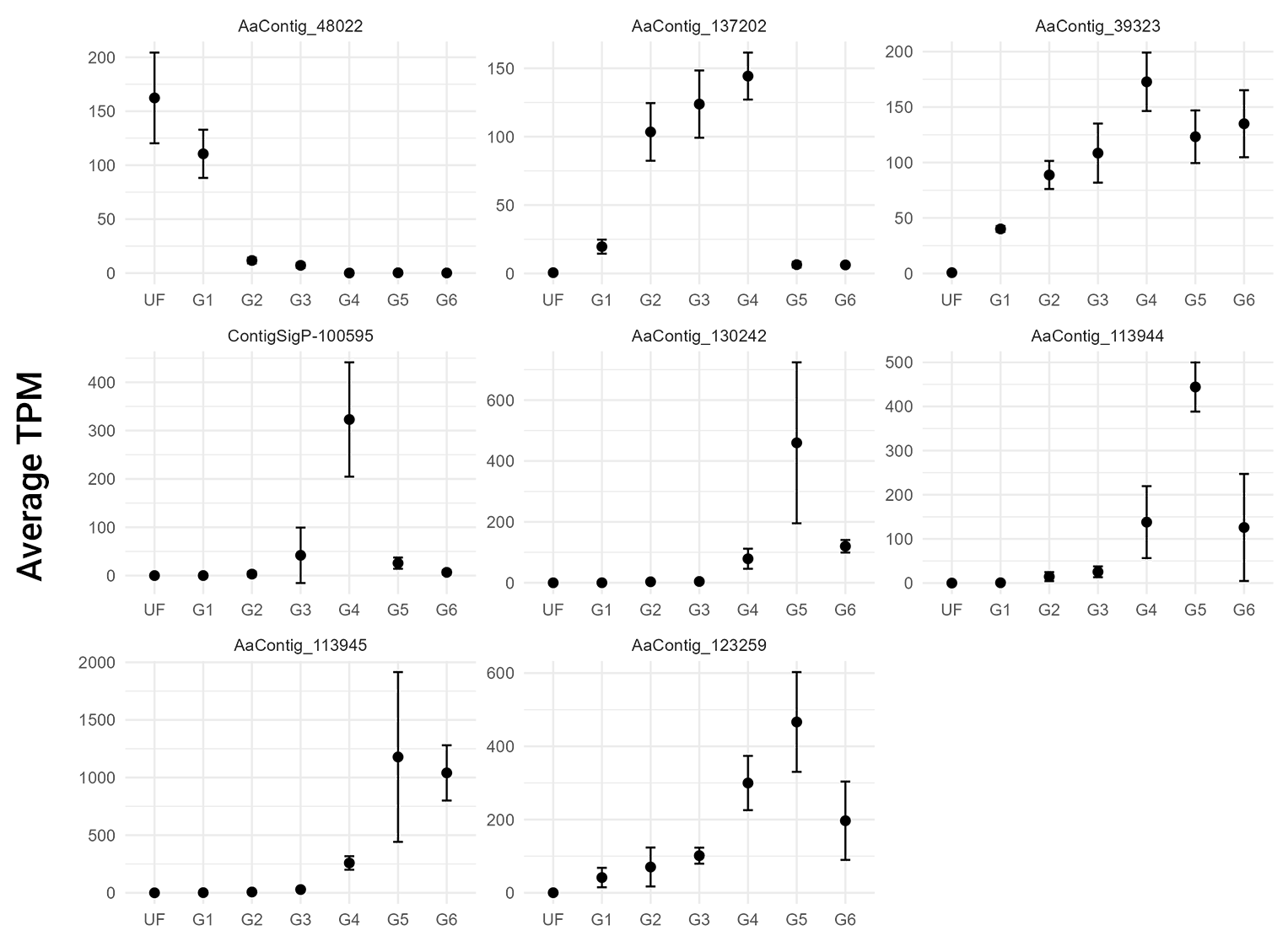
**

**Supplementary figure 3:** Expression profile of selected metalloproteases transcripts found differentially expressed in the salivary glands of *A. americanum* adult females at different feeding stages. Dots represent the average TPM and error bars the standard deviation of the mean.

**
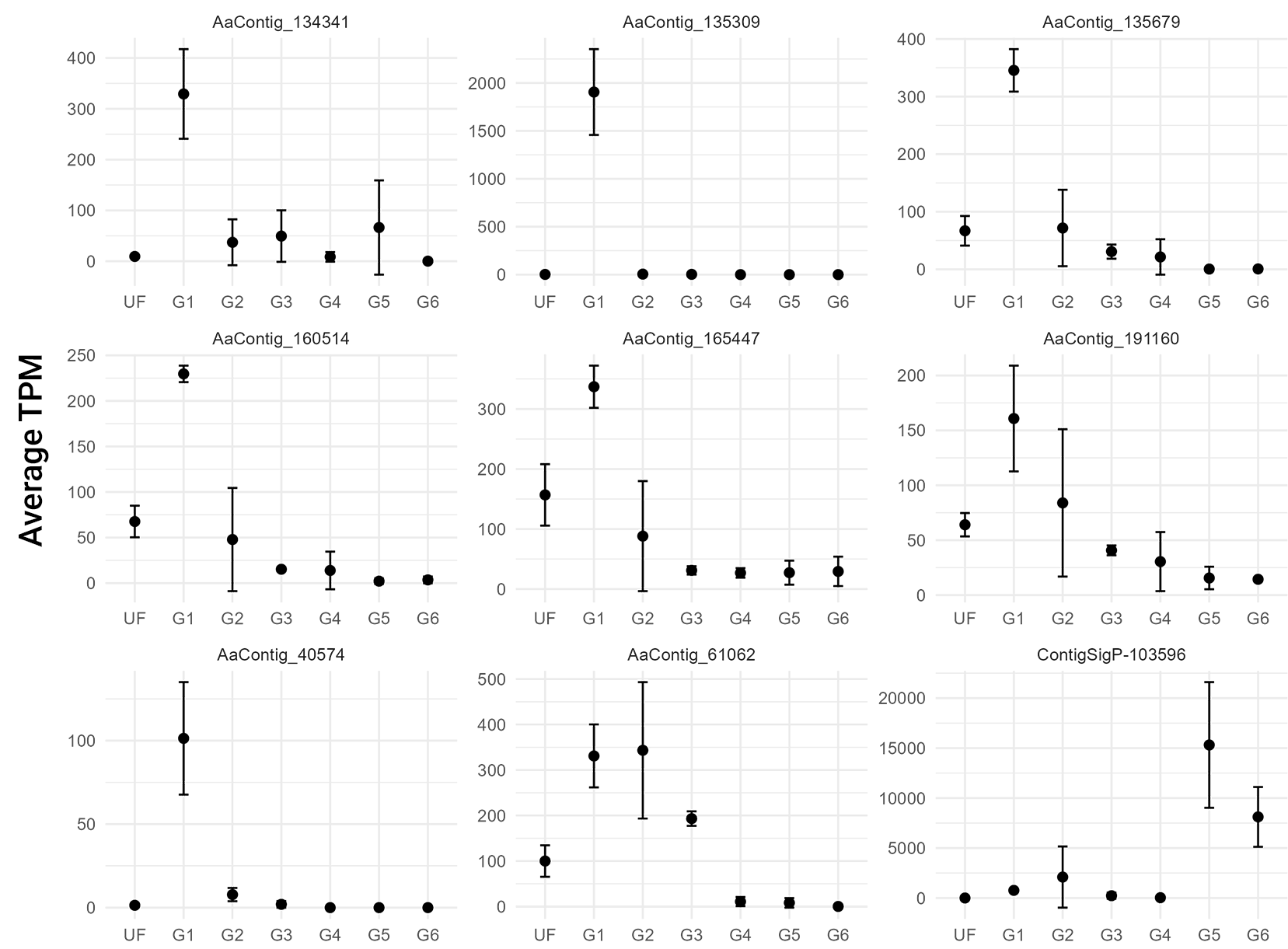
**

**Supplementary figure 4:** Expression profile of selected evasin-like transcripts found differentially expressed in the salivary glands of *A. americanum* adult females at different feeding stages. Dots represent the average TPM and error bars the standard deviation of the mean.
